# A transcriptome-defined TAM-rich phenotype identifies a macrophage-enriched, hypoxia-linked immune contexture in glioblastoma: multi-cohort transcriptomic validation and local histopathological correlation

**DOI:** 10.3389/fimmu.2026.1871268

**Published:** 2026-06-17

**Authors:** Guanpeng Li, Xiaohao Yan, Shenying Fang

**Affiliations:** 1 School of Public Health and Emergency Management, Southern University of Science and Technology, Shenzhen, China; 2 Sichuan Cancer Hospital, Chengdu, China; 3 Chengdu Huake Biological Research Center, Chengdu, China

**Keywords:** CAIX, CGGA, glioblastoma, hypoxia, immune microenvironment, TAM-rich phenotype, TCGA, transcriptomics

## Abstract

**Background:**

Tumor-associated macrophages (TAMs) are a major immune component of the glioblastoma microenvironment, but macrophage-oriented transcriptomic stratification remains difficult to reproduce across datasets and to validate at the tissue level.

**Methods:**

TCGA-GBM was used as the discovery cohort, and two independent CGGA cohorts (CGGA_693 and CGGA_325) were used for external validation. A continuous transcriptome-defined TAM score was constructed *a priori* as the mean log-scale expression of five macrophage-associated genes: CD163, MSR1, MRC1, CSF1R, and SPP1. Cohort-specific median dichotomization was used as a secondary categorical representation for visualization and group-level analyses. Differential expression, marker-level characterization, survival analysis, continuous-score sensitivity analyses, external immune benchmark analyses, and negative-control analyses were performed. Local histopathological correlation was assessed in a glioblastoma cohort from the Sichuan Cancer Hospital using immunohistochemistry and digital pathology for CD163, CD68, CD8, and CAIX.

**Results:**

In TCGA, the TAM-rich phenotype was associated with broad upregulation of macrophage- and myeloid-related genes. Across TCGA, CGGA_693, and CGGA_325, macrophage-associated markers, including CD163, MSR1, MRC1, CSF1R, SPP1, VSIG4, MARCO, and TREM1, were consistently increased, and the hypoxia-associated marker CA9 was also elevated. By contrast, CD8A, CXCL9, and CXCL10 showed only modest concordant increases. External immune benchmark analyses using xCell macrophage-related scores and published no-TAM5 macrophage/TAM signatures supported concordance with macrophage-related immune context, and random-panel and label-permutation analyses reduced concern that the associations reflected arbitrary five-gene scoring or random grouping. Survival association was modest and cohort-dependent. In the local cohort, CD163-defined macrophage-enriched tumors showed higher CD163-positive and CD68-positive macrophage density, greater CAIX-positive area, and a lower CD8/CD163 ratio, reflecting relative macrophage dominance rather than absolute loss of CD8-positive cells.

**Conclusions:**

A compact transcriptome-defined TAM-rich phenotype captures a biologically reproducible, macrophage-enriched and hypoxia-linked immune contexture in glioblastoma. It should be interpreted primarily as a quantitative immune-state classifier and tissue-correlated biological stratifier, rather than as a uniformly independent prognostic factor.

## 1 Introduction

Glioblastoma remains the most aggressive diffuse glioma in adults and continues to be associated with poor survival despite maximal safe resection, radiotherapy, and temozolomide-based treatment (1–3). The 2021 WHO classification further emphasized that adult-type diffuse gliomas should be interpreted within an integrated histomolecular framework, yet even with improved classification, glioblastoma still represents a disease in which biological heterogeneity and therapeutic resistance are major clinical problems (1, 4).

The limited efficacy of current immune-based strategies in glioblastoma has redirected attention toward the local tumor microenvironment rather than toward tumor cells alone (5, 6). Among non-malignant cell populations, microglia and macrophages constitute one of the dominant immune compartments in glioblastoma and are increasingly recognized as active contributors to tumor progression rather than passive bystanders (7–9). These cells influence invasion, angiogenesis, immune suppression, and therapeutic response, making them biologically attractive but analytically complex targets for translational study (7, 10, 11).

A major challenge is that glioblastoma-associated macrophages and microglia are not adequately captured by a simple M1/M2 framework (12). Single-cell studies have shown that glioma-associated myeloid cells differ by ontogeny, localization, and activation state, and that blood-derived macrophages and resident microglia can occupy distinct niches and transcriptional programs within the same tumor (13, 14). More recent work has further linked specific myeloid programs to mesenchymal tumor states and immunoregulatory signaling, highlighting markers such as MARCO and related macrophage-associated molecules as components of biologically meaningful glioblastoma ecosystems (15, 16). Together, these findings suggest that biologically useful macrophage-oriented phenotypes in glioblastoma should be compact enough to be portable across datasets, but also sufficiently grounded in known TAM biology to remain interpretable (9, 17).

Hypoxia provides a second layer of complexity that is difficult to ignore in glioblastoma biology. Hypoxic signaling contributes to tumor cell plasticity, metabolic adaptation, angiogenesis, and resistance to therapy, and it also shapes immune behavior within the tumor microenvironment (18, 19). Experimental and translational studies have suggested that hypoxia can reinforce suppressive myeloid states and intensify macrophage–glioma interactions (20, 21). CAIX, a downstream hypoxia-associated marker, has attracted interest not only as a metabolic indicator but also as a feature of spatially and functionally distinct tumor regions in glioblastoma (22, 23). These observations raise the possibility that macrophage-rich and hypoxia-linked programs may co-segregate in a biologically meaningful subset of glioblastomas rather than representing unrelated background features (24, 25).

At the same time, the relationship between myeloid enrichment and T-cell status in glioblastoma is more nuanced than a simple immune-hot versus immune-cold dichotomy. T-cell dysfunction, exhaustion, and ineffective effector activity are well-recognized features of malignant gliomas (26). However, several studies suggest that poor immune control in glioblastoma may reflect suppressive myeloid dominance, spatial exclusion, and functional impairment rather than complete absence of lymphocytes (27, 28). This distinction is important, because a macrophage-rich tumor does not necessarily have to be globally lymphocyte-depleted. A phenotype that preserves detectable T-cell-related signals while remaining strongly myeloid-biased may be more consistent with real glioblastoma immunobiology than a purely immune-cold label (26, 29).

Several macrophage-associated markers have already shown biological and translational relevance in glioma. CD163 has been linked to higher glioma grade, IDH-wildtype status, mesenchymal biology, and poorer clinical context (30). CSF1R remains one of the best-established myeloid-regulatory axes in glioblastoma, with preclinical evidence that altering CSF1R signaling can remodel macrophage behavior and slow glioma progression (31). SPP1 has emerged as a particularly informative marker of immunoregulatory macrophage programs in glioma and other tumors, while MRC1 and MSR1 remain widely used indicators of macrophage-rich, scavenger-oriented states (12, 32, 33). Yet, despite the abundance of literature on glioblastoma myeloid biology, there is still a gap between complex mechanistic insight and practical transcriptomic phenotyping that can be reproduced across public cohorts and meaningfully anchored to local histopathology (9, 34).

Against this background, we designed the present study to characterize a compact transcriptome-defined TAM-rich phenotype in glioblastoma and to test whether it is reproducible across independent transcriptomic cohorts and recognizable as a parallel tissue-level macrophage-enriched contexture. Rather than building a broad deconvolution model or an overparameterized classifier, we focused on a predefined 5-gene macrophage-oriented panel composed of CD163, MSR1, MRC1, CSF1R, and SPP1, selected for biological relevance and stable representation in bulk transcriptomic datasets. We hypothesized that this phenotype would identify a macrophage-enriched state that is biologically consistent across datasets, linked to hypoxia-related features, and better interpreted as a macrophage-dominant immunoregulatory niche than as a purely immune-cold state. To test this, we used TCGA-GBM as the discovery cohort, CGGA_693 and CGGA_325 as external validation cohorts, and an independent local histopathological cohort from the Sichuan Cancer Hospital for tissue-level correlation.

## 2 Materials and methods

### 2.1 Study design and overall analytical framework

This study was designed as a multi-cohort transcriptomic investigation of a macrophage-oriented immune phenotype in glioblastoma. TCGA-GBM was used as the discovery cohort for phenotype definition and initial biological characterization. Two independent CGGA transcriptomic cohorts, CGGA_693 and CGGA_325, were then used for external transcriptomic validation and robustness assessment. Finally, tissue-level translational correlation was performed in a local histopathological cohort from the Sichuan Cancer Hospital.

### 2.2 Public transcriptomic cohorts and data preprocessing

Public transcriptomic datasets were obtained from TCGA and CGGA. For TCGA-GBM, gene-level RNA-seq count files were downloaded from the GDC portal. The unstranded count column was extracted from each file and mapped to patient-level identifiers using the GDC sample sheet. Gene symbols with missing annotations were removed, and duplicated gene symbols were resolved by retaining the record with the highest mean expression across samples. Lowly expressed genes were filtered by requiring CPM > 1 in at least 10% of samples, with a minimum of 10 samples. Library-size normalization was performed using the trimmed mean of M values method implemented in *edgeR*, and normalized log2 counts per million values were generated with a prior count of 1.

Two CGGA transcriptomic cohorts, CGGA_693 and CGGA_325, were used as external validation datasets. For both CGGA cohorts, gene-level FPKM matrices were imported and transformed as log2(FPKM + 1) before downstream analysis. Eligible cases were restricted to glioblastoma samples with available gene-expression data and matched clinical information. Samples were excluded if they lacked analyzable overall survival time, survival status, or matched transcriptomic identifiers. Expression matrices were harmonized at the gene-symbol level within each cohort. All phenotype definitions and downstream analyses were performed separately within each cohort, without merging expression matrices across platforms, to reduce scaling artifacts related to data source and expression-unit differences.

### 2.3 Definition of the transcriptome-defined TAM-rich phenotype

The primary exposure of interest was a transcriptome-defined TAM-rich phenotype derived from a predefined compact macrophage-oriented panel consisting of CD163, MSR1, MRC1, CSF1R, and SPP1. These genes were selected *a priori* based on recurrent use in glioma-associated macrophage literature, biological relevance to macrophage-rich and immunoregulatory states in glioma, and stable representation across the bulk transcriptomic datasets used in the present study. The intention was to define a portable and interpretable macrophage-oriented transcriptomic construct rather than to claim a complete or canonical macrophage subtype program.

For each cohort, a continuous TAM score was calculated as the arithmetic mean of the five log-scale gene expression values and was treated as the primary quantitative representation of the phenotype. Tumors were dichotomized at the cohort-specific median score into TAM-rich and TAM-low groups for visualization, descriptive group comparisons, and secondary categorical survival analyses. Exact cohort-specific median cutoffs, score distributions, interquartile ranges, ranges, and component-gene expression distributions are reported in **Supplementary Table 6** and **Supplementary Figure 5**. Because TCGA and CGGA datasets were generated from different expression platforms and scales, raw cutoffs were not transferred directly across cohorts. As a cross-platform sensitivity analysis, Cox models were additionally fitted using the within-cohort z-score standardized continuous 5-gene score, estimating the hazard ratio per 1-SD increase in score.

### 2.4 Differential expression analysis in the discovery cohort

Differential expression analysis between tumors with the TAM-rich phenotype and those with the TAM-low phenotype was performed in the TCGA discovery cohort using the *limma* framework, which fits linear models to gene-expression data and uses empirical Bayes moderation to improve variance estimation across genes (35). Differentially expressed genes were ranked primarily by adjusted *P* value and secondarily by effect size, and false discovery rate (FDR) control was applied using the Benjamini–Hochberg procedure. Volcano plots and ranked gene tables were generated to summarize the transcriptomic shifts associated with the phenotype. Representative marker-level expression plots were then produced for macrophage-associated, T-cell-related, and hypoxia-related genes.

### 2.5 Marker-oriented biological characterization

To provide an interpretable biological overview, a focused marker panel was examined across cohorts. Markers were grouped into three conceptual categories: macrophage-associated markers, hypoxia-associated markers, and T-cell-related markers. The five score-defining genes were CD163, MSR1, MRC1, CSF1R, and SPP1. Additional non-defining supportive macrophage-oriented markers included VSIG4, MARCO, and TREM1. The hypoxia-associated transcriptomic marker was represented by CA9, which encodes carbonic anhydrase IX (CAIX). In the local histopathological cohort, CAIX protein expression was assessed by immunohistochemistry. T-cell-related features were represented by CD8A, CXCL9, and CXCL10. Heatmaps and boxplots were used to visualize group-wise expression patterns. These marker-level, immune-score, local IHC, and correlation analyses were predefined targeted exploratory analyses intended for biological characterization rather than additional phenotype construction or formal high-dimensional hypothesis discovery.

### 2.6 External transcriptomic validation

External validation was performed independently in CGGA_693 and CGGA_325 using the same phenotype definition and the same direction of comparison as in the discovery cohort. For each validation cohort, the predefined 5-gene score was computed, cohort-specific median dichotomization was applied, and the same marker-oriented and clinical analyses were performed. CGGA_693 was treated as the primary external validation cohort, whereas CGGA_325 served as a secondary robustness dataset. To summarize cross-cohort consistency, log2 fold changes for selected markers were extracted and displayed in a compact cross-dataset heatmap. Cohort-wise survival associations were additionally summarized using forest-style presentation.

### 2.7 Supportive immune infiltration, external benchmark, and negative-control analyses

We defined the term “purely immune-cold” to describe tumors with broadly low immune-related activity, particularly including low T-cell-related signals. First, MCP-counter was applied to estimate abundance scores for monocytic lineage, T cells, and cytotoxic lymphocytes from bulk transcriptomic data. Second, sample-wise enrichment scores were computed using a GSVA/ssGSEA-style approach (36) for three supportive immune programs: macrophage-related, myeloid-related, and T-cell-related signatures. To reduce direct circularity, these signatures were defined without the five core phenotype genes (CD163, MSR1, MRC1, CSF1R, and SPP1) and without CD8A, CXCL9, or CXCL10. Full gene lists are provided in **Supplementary Table 4**. Because the remaining genes are still lineage-related and co-regulated, these analyses were interpreted as supportive immune-context analyses rather than fully independent validation.

Additional external immune benchmark and negative-control analyses were performed to address potential circularity. xCell was used to generate macrophage-related benchmark scores, including Macrophages, Macrophages M1, and Macrophages M2 (37). In addition, published macrophage/TAM-related benchmark signatures were evaluated by ssGSEA, with the five TAM-score-defining genes excluded from published benchmark signatures where applicable. Associations between the continuous TAM5 score and benchmark scores were assessed using Spearman correlation, and benchmark-score differences between TAM-low and TAM-rich tumors were summarized using median differences. Two negative-control analyses were performed: 1,000 expression-matched random 5-gene panels excluding the TAM5 genes, and phenotype-label permutation while preserving group sizes. Empirical P values were calculated as (1 + number of null statistics at least as extreme as the observed statistic)/(1 + number of valid random panels or permutations).

### 2.8 Local histopathological cohort

For tissue-level histopathological correlation, an independent retrospective glioblastoma cohort was assembled from the Sichuan Cancer Hospital. A total of 58 eligible cases with available formalin-fixed paraffin-embedded (FFPE) tumor tissue and corresponding clinicopathological information were included. Cases were restricted to pathologically confirmed glioblastoma with sufficient viable tumor tissue for immunohistochemical staining and digital pathology analysis. Recurrent tumors, severely compromised archived material, specimens with extensive necrosis precluding evaluation, or specimens with inadequate tissue quality were excluded.

Because matched transcriptomic data were not available for the local FFPE cohort, this cohort was used as a tissue-level histopathological correlate rather than as a cohort for transcriptomic phenotype assignment. Local cases were stratified into macrophage-low and CD163-defined macrophage-enriched groups according to the median case-level CD163-positive macrophage density. This grouping was used only for tissue-level correlation and was interpreted as a histopathological analogue of the macrophage-enriched state observed in the transcriptomic cohorts.

The local histopathological correlation study was approved by the Ethics Committee of the Sichuan Cancer Hospital (approval no. sch23091). The requirement for informed consent was waived because archived specimens and de-identified data were used.

### 2.9 Immunohistochemistry and representative marker selection

Immunohistochemical staining was performed on 4-microm FFPE sections using protocols adapted from recent immunohistochemistry studies using FFPE tissue sections and chromogenic or multiplex immune-marker detection (38–41). Briefly, sections were deparaffinized in xylene, rehydrated through graded ethanol, subjected to heat-induced antigen retrieval, blocked for endogenous peroxidase activity and nonspecific background, incubated with primary antibodies against CD163, CD68, CD8, and CAIX under optimized laboratory-standard conditions, and then developed using an HRP-based secondary detection system with DAB chromogen followed by hematoxylin counterstaining. CD163 and CD68 were used to characterize macrophage-rich tissue features, CD8 was used to assess cytotoxic T-cell presence, and CAIX was used to represent hypoxia-related tissue context.

The same pre-analytical and staining workflow was applied across all cases for a given marker. Negative-control slides without primary antibody and routine review of staining intensity, background, and tissue preservation were used for quality control. Slides with severe tissue folding, extensive detachment, marked nonspecific background staining, or technically inadequate staining were excluded before quantitative analysis. Representative images were selected from serial sections after quantitative analysis. To reduce selection bias, representative cases were chosen from cases with values close to the median of their corresponding tissue-level group and with technically adequate staining quality. Higher-magnification insets were included to illustrate cellular staining patterns.

For each marker, stained slides were reviewed for staining quality before digital analysis. Slides with severe tissue folding, extensive detachment, marked non-specific background staining, or technically inadequate staining were excluded from quantitative assessment. Representative images were selected from serial sections of one macrophage-low representative case and one CD163-defined macrophage-enriched representative case after completion of quantitative analysis. To reduce selection bias, representative cases were chosen from cases with quantitative values close to the median of their corresponding tissue-level group and with technically adequate staining quality. Higher-magnification insets were included to illustrate cellular staining patterns.

### 2.10 Digital pathology quantification

Digital pathology quantification was performed on scanned immunohistochemical slides using QuPath version 0.5.1. For each case, three non-overlapping regions of interest (ROIs) were selected whenever sufficient evaluable tumor tissue was available. Each ROI was standardized to approximately 1.0 mm^2^. When the available tumor area was limited, all evaluable ROIs meeting quality criteria were included, and the number of analyzed ROIs was recorded.

ROIs were selected from viable tumor-rich areas by observers blinded to group assignment and clinical outcome. To capture intratumoral heterogeneity while avoiding artefact-driven measurements, ROI selection preferentially included morphologically representative tumor areas and, when present, regions adjacent to but not within necrosis. Large necrotic cores, hemorrhage, tissue folds, crushed regions, large vessels, section edges, and areas with obvious staining artefacts were excluded. For serial sections stained with different markers, corresponding anatomical regions were selected as closely as possible to maintain comparability across CD163, CD68, CD8, and CAIX staining.

For CD163, CD68, and CD8, positive cells were quantified as positive cell density and expressed as cells/mm² within the annotated tumor ROI. Case-level values were calculated by averaging ROI-level measurements across all evaluable ROIs from the same case. For CAIX, staining was quantified as the percentage of CAIX-positive area within the annotated tumor ROI, and case-level CAIX-positive area was calculated as the mean percentage across ROIs. To capture the balance between T-cell and macrophage compartments, the CD8/CD163 ratio was calculated at the case level as:


CD8/CD163 ratio = (CD8−positive cell density + 0.01)/(CD163−positive macrophage density + 0.01)

where 0.01 was added as a small pseudocount to avoid undefined values in cases with extremely low cell counts.

The primary tissue-level quantitative outputs were CD163-positive macrophage density, CD68-positive macrophage density, CD8-positive T-cell density, CAIX-positive area percentage, and the CD8/CD163 ratio. These metrics were compared between tissue-level TAM-low and TAM-rich local tumors. Spearman correlation analyses were further used to evaluate the relationships between CD163-positive macrophage density and CD68-positive macrophage density, between CD163-positive macrophage density and CAIX-positive area, and between CAIX-positive area and the CD8/CD163 ratio.

### 2.11 Sensitivity analyses of phenotype robustness

Three complementary sensitivity analyses were prespecified to examine robustness of the phenotype definition. First, leave-one-gene-out analyses were performed by removing each of the five component genes in turn and recalculating the phenotype score. Second, a specific 4-gene panel excluding SPP1 was examined because of the biological relevance of SPP1 to macrophage functional state. Third, continuous-score sensitivity analyses were conducted using the standardized phenotype score rather than relying only on median dichotomization.

For each reformulation, two metrics were calculated relative to the original 5-gene phenotype (1): the agreement in binary phenotype assignment and (2) the Spearman correlation between recalculated and original continuous scores. Cox models were then repeated using both binary and continuous formulations. These analyses were designed to test whether the phenotype behaved as a coherent transcriptomic construct rather than as a single-marker artifact.

### 2.12 Statistical analysis

All analyses were conducted in R using the final project scripts and cohort-specific harmonized datasets. Continuous variables were summarized as median (interquartile range) unless otherwise stated, and categorical variables as n (%). Wilcoxon rank-sum tests and Fisher’s exact tests were used for baseline group comparisons. Differential expression was estimated using limma with empirical Bayes moderation. Kaplan–Meier curves and log-rank tests were used for unadjusted visualization of overall survival according to phenotypic status. Cox proportional hazards models were used to estimate hazard ratios and 95% confidence intervals. Cox models were adjusted for age and sex when both variables were available in the corresponding cohort-specific clinical dataset. The same adjustment strategy was applied to binary phenotype models and continuous-score sensitivity models. Orthogonal immune infiltration analyses used MCP-counter and GSVA-based sample-wise scoring. Correlations were assessed using Spearman’s rank correlation, consistent with the final figure outputs. All statistical tests were two-sided, and *P* values<0.05 were considered statistically significant unless otherwise specified. For transcriptome-wide differential expression, multiple testing was controlled using FDR adjustment. Marker-level comparisons, immune-score comparisons, local IHC analyses, and correlation analyses were treated as targeted exploratory analyses and were reported with nominal two-sided P values without additional multiplicity correction unless otherwise specified. External benchmark score correlations were additionally summarized with Benjamini-Hochberg FDR values, and random-panel and label-permutation negative controls were evaluated using empirical P values.

## 3 Results

### 3.1 Study overview and cohort structure

**Figure 1:**
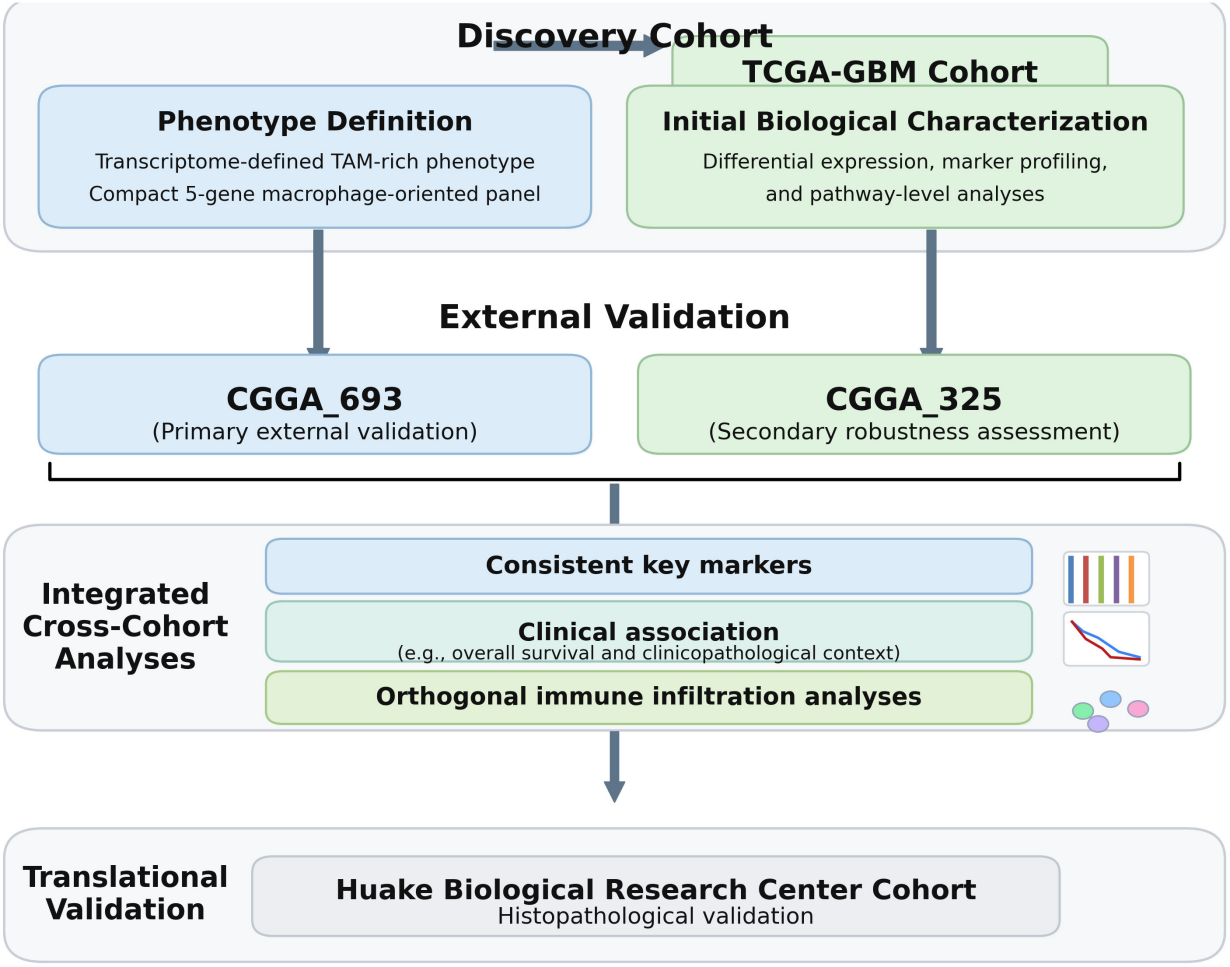
Study design and analytical workflow. This study was designed as a multi-cohort transcriptomic investigation of a tumor-associated macrophage-enriched state in glioblastoma. TCGA-GBM served as the discovery cohort for phenotype definition and initial biological characterization. External validation was subsequently performed in two independent CGGA transcriptomic cohorts, with CGGA_693 used as the primary external validation cohort and CGGA_325 used for secondary robustness assessment. Cross-cohort analyses then evaluated consistency of key markers, clinical association, supportive immune infiltration patterns, external benchmark analyses, and negative controls. Local histopathological correlation was performed in a glioblastoma cohort from the Sichuan Cancer Hospital.

The overall study design is shown in **Figure 1**. TCGA-GBM was used as the discovery cohort for phenotype definition and initial biological characterization, followed by external validation in two independent CGGA transcriptomic cohorts. Cross-cohort analyses then focused on consistency of key markers, clinical association, supportive immune infiltration patterns, external benchmark analyses, and negative controls. A local histopathological cohort from the Sichuan Cancer Hospital was used for tissue-level translational validation.

In the TCGA discovery cohort, 285 transcriptome-matched cases were available for analysis. Baseline characteristics stratified by the transcriptome-defined TAM-rich phenotype are summarized in **Table 1**. Age, sex distribution, overall survival time, and vital status did not differ significantly between the TAM-low and TAM-rich groups (all *P* > 0.05), indicating that the two phenotypic groups were broadly comparable at baseline.

**Table 1:** Baseline characteristics of the TCGA-GBM discovery cohort according to the transcriptome-defined TAM-rich phenotype.

Variable	Level	Overall	TAM-low	TAM-rich	*P* value
Age, years		60.0 (50.0–69.0)	59.0 (49.0–68.8)	61.0 (52.5–69.0)	0.119
Overall survival time, days		382.0 (164.0–585.0)	394.5 (169.0–636.8)	360.0 (160.5–562.5)	0.483
Sex	female	105 (36.8%)	58 (40.8%)	47 (32.9%)	0.178
	male	180 (63.2%)	84 (59.2%)	96 (67.1%)	
Vital status	Alive	53 (18.6%)	29 (20.4%)	24 (16.8%)	0.450
	Dead	232 (81.4%)	113 (79.6%)	119 (83.2%)	

Continuous variables are presented as median (IQR), and categorical variables are presented as n (%). P values were calculated using the Wilcoxon rank-sum test for continuous variables and Fisher’s exact test for categorical variables, as appropriate.

To support interpretation of the external validation and tissue-level analyses, baseline characteristics of CGGA_693 and CGGA_325 are summarized in **Supplementary Table 1, 2**, respectively. Baseline and digital pathology characteristics of the local histopathological cohort are summarized in **Supplementary Table 3**.

### 3.2 Identification of a transcriptome-defined TAM-rich phenotype in the TCGA discovery cohort

**Figure 2:**
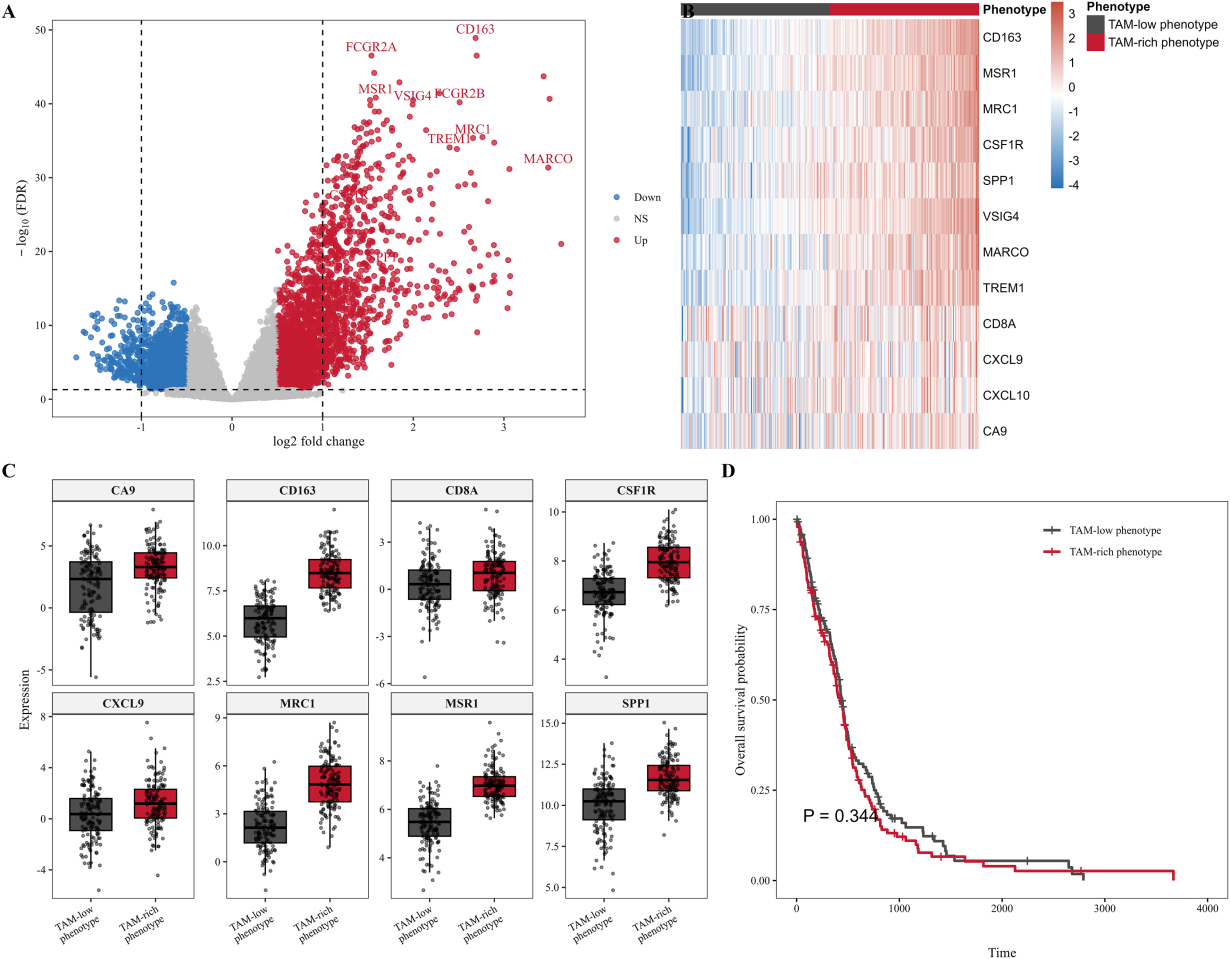
Identification and biological characterization of the transcriptome-defined TAM-rich phenotype in the TCGA discovery cohort. **(A)** Volcano plot of differentially expressed genes between tumors with the TAM-rich phenotype and those with the TAM-low phenotype in TCGA. Selected macrophage-associated genes are highlighted. **(B)** Heatmap showing the expression patterns of representative macrophage-, T-cell-, and hypoxia-related markers across the two phenotypic groups. **(C)** Boxplots comparing representative marker expression between groups. Canonical macrophage-associated markers, including CD163, MSR1, MRC1, CSF1R, and SPP1, were markedly increased in tumors with the TAM-rich phenotype. **(D)** Kaplan–Meier analysis of overall survival according to phenotypic status in the TCGA cohort, shown as an exploratory clinical association rather than the primary basis of phenotype definition.

Using the predefined 5-gene macrophage-oriented panel, tumors in TCGA were stratified into TAM-low and TAM-rich phenotypes for descriptive comparison. Differential expression analysis demonstrated broad transcriptomic differences between the two groups (**Figure 2A**). Upregulation of the score-defining genes was expected given the score definition; therefore, biological interpretation focused not only on these genes but also on non-defining macrophage- or myeloid-related genes, including FCGR2A, VSIG4, FCGR2B, SIGLEC9, HK3, IL10RA, FPR2, KYNU, F13A1, MS4A4A, IL7R, IL2RA, and MAP3K8, which further supported a myeloid- and immune-regulatory transcriptomic program (**Table 2**).

**Table 2:** Top differentially expressed genes associated with the TAM-rich phenotype in the TCGA discovery cohort.

Gene	logFC	AveExpr	t	*P* value	FDR
CD163	2.687	7.182	19.383	<0.001	<0.001
FPR2	2.699	0.769	18.633	<0.001	<0.001
FCGR2A	1.538	5.801	18.597	<0.001	<0.001
KYNU	1.568	2.373	17.926	<0.001	<0.001
F13A1	3.438	5.731	17.774	<0.001	<0.001
MS4A4A	1.847	4.455	17.532	<0.001	<0.001
IL7R	2.287	1.963	17.112	<0.001	<0.001
MSR1	1.586	6.225	16.933	<0.001	<0.001
IL2RA	3.503	1.007	16.876	<0.001	<0.001
HK3	1.998	2.864	16.806	<0.001	<0.001
SIGLEC9	1.523	3.371	16.808	<0.001	<0.001
FCGR2B	2.511	2.691	16.716	<0.001	<0.001
VSIG4	1.993	7.129	16.623	<0.001	<0.001
IL10RA	1.526	5.205	16.586	<0.001	<0.001
MAP3K8	1.621	3.446	16.345	<0.001	<0.001

Genes were ranked primarily by adjusted P value and secondarily by absolute log2 fold change. Differential expression was estimated using limma.

Heatmap visualization of representative markers showed a coherent shift toward higher expression of macrophage-associated genes in the TAM-rich group (**Figure 2B**). In parallel, boxplot analyses confirmed that canonical macrophage-related markers, including CD163, CSF1R, MRC1, MSR1, and SPP1, were increased in tumors with the TAM-rich phenotype (**Figure 2C**). The hypoxia-related marker CA9 was also higher in the TAM-rich group. By contrast, T-cell-related markers such as CD8A and CXCL9 were increased only modestly, suggesting that the phenotype was not simply equivalent to a globally lymphocyte-depleted state.

Kaplan–Meier analysis in the TCGA discovery cohort showed no significant unadjusted separation in overall survival between the two phenotypic groups (**Figure 2D**), indicating that the primary value of this phenotype in the discovery cohort lay in biological characterization rather than strong standalone prognostic discrimination.

### 3.3 Cross-cohort validation showed consistent enrichment of macrophage-associated markers

**Figure 3:**
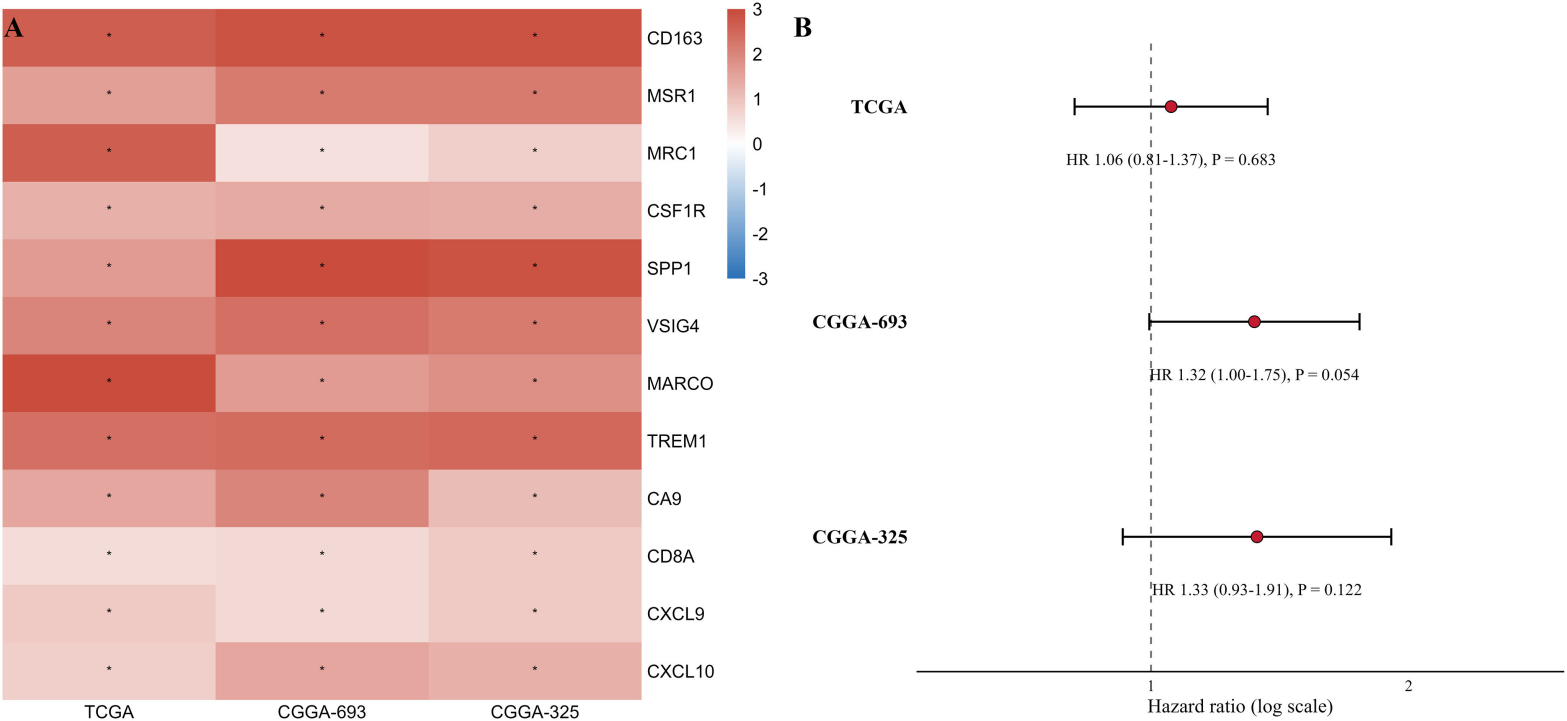
Cross-cohort validation of the transcriptome-defined TAM-rich phenotype. **(A)** Heatmap showing log2 fold changes of selected macrophage-associated, hypoxia-related, and inflammatory markers in TAM-rich versus TAM-low tumors across TCGA, CGGA_693, and CGGA_325. Log2 fold changes were obtained from independent cohort-specific differential-expression tests. Cell labels indicate statistical significance after multiple-testing correction within each cohort-specific comparison: *FDR< 0.05; ns, FDR >= 0.05. For visualization, the heatmap color scale was truncated at log2 fold change values of -3 and +3; exact values are provided in **Table 3**. **(B)** Forest plot showing Cox proportional hazards models for overall survival using the recalculated five-gene TAM score-derived binary classification, defined by cohort-specific median cutoffs. Models were adjusted for available age and sex variables within each cohort. Points indicate hazard ratios, horizontal bars indicate 95% confidence intervals, and the dashed vertical line indicates HR = 1. The binary classification was used as a secondary categorical representation of the continuous score.

Cross-cohort comparison of selected markers demonstrated a directionally consistent biological pattern across TCGA, CGGA_693, and CGGA_325, although effect-size heterogeneity was present for some markers (**Figure 3A**; **Table 3**). Log2 fold changes in **Figure 3A** were obtained from independent cohort-specific differential-expression tests, and cell labels indicate whether the corresponding marker-level comparison reached FDR< 0.05. For visualization, the heatmap color scale was truncated at ±3, whereas exact log2 fold changes and FDR values are reported in **Table 3**. Macrophage-associated genes were upregulated in tumors with the TAM-rich phenotype across all three cohorts, including the five defining genes CD163, MSR1, MRC1, CSF1R, and SPP1, as well as non-defining supportive markers VSIG4, MARCO, and TREM1. MRC1 preserved a positive direction in all cohorts but showed heterogeneous effect sizes, with a larger shift in TCGA than in CGGA_693 or CGGA_325. The hypoxia-associated marker CA9 was also consistently increased. While MRC1 effect sizes varied across cohorts – likely due to platform differences, tumor purity, or sample composition – the direction of effect was uniformly positive, supporting a reproducible macrophage-enriched pattern.

**Table 3:** Cross-cohort consistency of selected markers associated with the transcriptome-defined TAM-rich phenotype.

Marker	Category	TCGA	CGGA-693	CGGA-325
CD163	Macrophage-associated	2.69	2.86	2.88
MSR1	Macrophage-associated	1.59	2.19	2.20
MRC1	Macrophage-associated	2.66	0.52	0.81
CSF1R	Macrophage-associated	1.29	1.41	1.34
SPP1	Macrophage-associated	1.66	3.55	2.87
VSIG4	Macrophage-associated	1.99	2.39	2.17
MARCO	Macrophage-associated	3.49	1.65	1.82
TREM1	Macrophage-associated	2.40	2.46	2.47
CA9	Hypoxia-associated	1.46	2.01	1.13
CD8A	T cell-related	0.59	0.62	0.89
CXCL9	T cell-related	0.86	0.63	0.89
CXCL10	T cell-related	0.81	1.47	1.29

Positive log2 fold change values indicate higher expression in tumors with the TAM-rich phenotype. Markers were selected a priori to represent macrophage-associated, hypoxia-associated, and T cell-related programs.

In contrast, T-cell-related markers showed only modest concordant elevation. CD8A was positively shifted in all three cohorts (0.59, 0.62, and 0.89), as were CXCL9 (0.86, 0.63, and 0.89) and CXCL10 (0.81, 1.47, and 1.29). This pattern was directionally consistent with the discovery-cohort marker plots and argued against interpreting the TAM-rich phenotype as a purely immune-cold state.

### 3.4 Clinical association across cohorts was modest and cohort-dependent

Across cohorts, the transcriptome-defined TAM-rich phenotype showed modest and cohort-dependent clinical association (**Figure 3B**; **Supplementary Tables 5**, **S6**). In adjusted binary Cox models, the original 5-gene phenotype was not statistically significant in TCGA (HR 1.06, 95% CI 0.81-1.37, P = 0.683), CGGA_693 (HR 1.32, 95% CI 1.00-1.75, P = 0.054), or CGGA_325 (HR 1.33, 95% CI 0.93-1.91, P = 0.122). Within-cohort z-score standardized continuous-score Cox models showed modest positive associations in CGGA_693 (HR 1.14, 95% CI 1.00-1.31, P = 0.048) and CGGA_325 (HR 1.21, 95% CI 1.01-1.46, P = 0.044), whereas the association did not reach statistical significance in TCGA (HR 1.10, 95% CI 0.97-1.26, P = 0.137).

The continuous score distributions and exact cohort-specific median cutoffs are reported in **Supplementary Table 6** and **Supplementary Figure 5**. The median cutoffs were 7.06 in TCGA, 3.98 in CGGA_693, and 5.26 in CGGA_325. Because raw expression scales differ across platforms, these cutoffs were used within cohorts and were not transferred directly between datasets. Taken together, these results suggest that the phenotype is biologically robust across cohorts, whereas its survival association is modest, context-dependent, and better interpreted as exploratory rather than as evidence of a uniformly independent prognostic factor.

### 3.5 Supportive immune infiltration and external benchmark analyses supported a macrophage-dominant immune contexture

**Figure 4:**
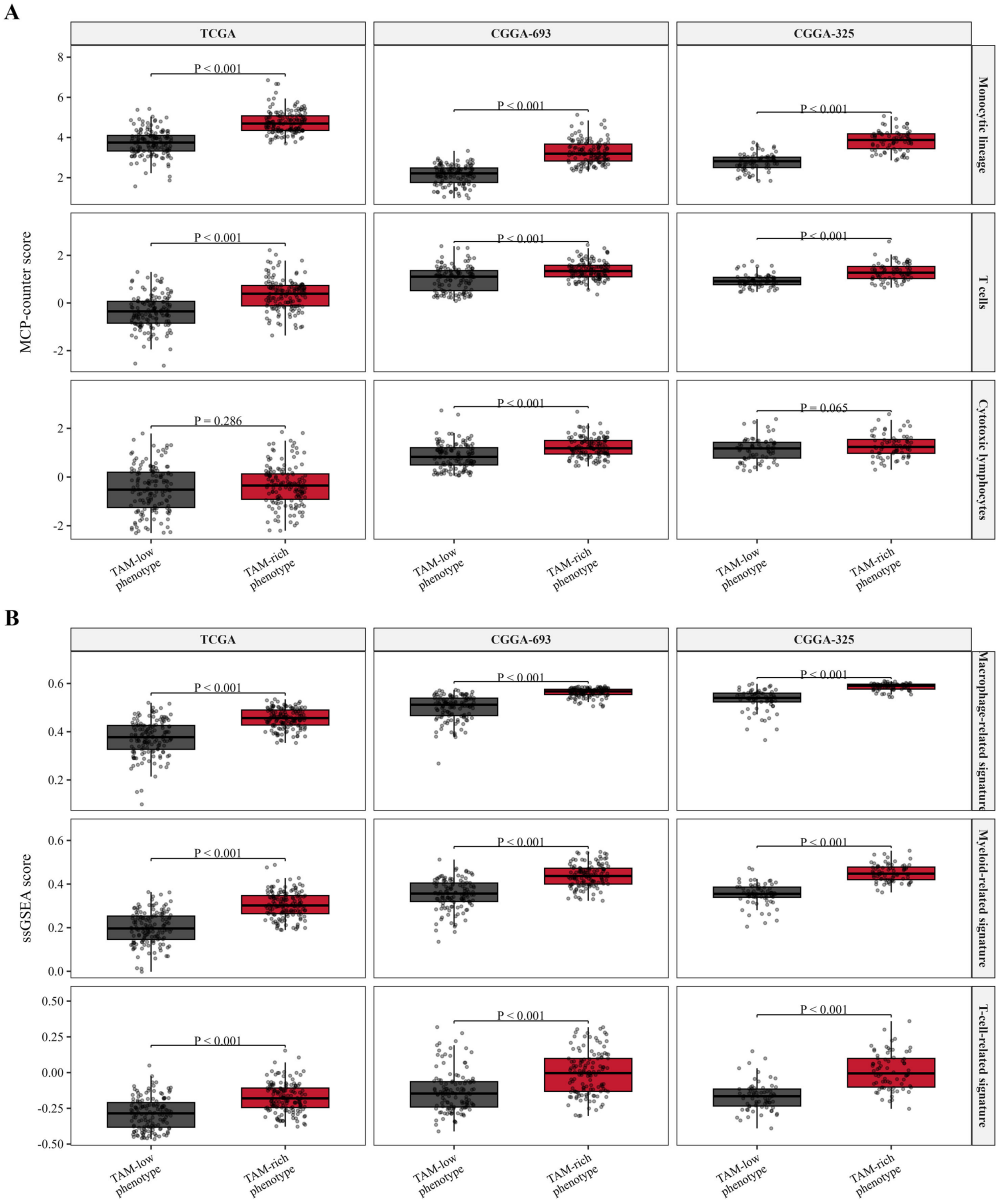
Supportive immune infiltration analyses across discovery and validation cohorts. **(A)** MCP-counter scores for monocytic lineage, T cells, and cytotoxic lymphocytes stratified by phenotypic status in TCGA, CGGA_693, and CGGA_325. P values were calculated using two-sided Wilcoxon rank-sum tests comparing TAM-low and TAM-rich tumors within each dataset and score type. **(B)** Supportive ssGSEA/GSVA scores for macrophage-, myeloid-, and T-cell-related signatures across the same cohorts. These gene sets were intentionally defined without the core phenotype-defining macrophage genes (CD163, MSR1, MRC1, CSF1R, and SPP1) and without CD8A/CXCL9/CXCL10 to reduce direct circularity. Because the remaining genes remain lineage-related and co-regulated, these analyses are interpreted as supportive immune-context analyses rather than fully independent validation.

To examine whether the phenotype was accompanied by broader immune-context changes beyond direct expression of the five defining genes, supportive immune infiltration analyses were performed across all three cohorts (**Figure 4**; **Supplementary Figure 3**). MCP-counter showed that tumors with the TAM-rich phenotype had consistently higher monocytic lineage scores in TCGA, CGGA_693, and CGGA_325. T-cell and cytotoxic lymphocyte scores were not globally suppressed and were, in several cohort-specific comparisons, slightly higher in the TAM-rich group. Nominal P values are displayed in **Figure 4** to support transparency for these targeted exploratory immune-score comparisons.

A similar pattern was observed using supportive ssGSEA/GSVA signatures intentionally defined without the core phenotype genes and without CD8A/CXCL9/CXCL10; the full gene lists are provided in **Supplementary Table 4**. Across cohorts, the TAM-rich phenotype was associated with higher macrophage-related and myeloid-related enrichment scores, whereas T-cell-related signature scores were not uniformly reduced. Because these signatures remain lineage-related and co-regulated, they are interpreted as supportive immune-context analyses rather than fully independent validation.

To further reduce potential circularity, we added external immune benchmark and negative-control analyses (**Supplementary Table 7**; **Supplementary Figures 7**–**9**). xCell macrophage-related scores were consistently higher in TAM-rich tumors and strongly correlated with the continuous TAM5 score across cohorts. For the xCell macrophage score, Spearman correlations with the TAM5 score were 0.87 in TCGA, 0.85 in CGGA_693, and 0.92 in CGGA_325, with all associations remaining significant after multiple-testing correction. Published macrophage/TAM benchmark signatures excluding the five TAM-score-defining genes where applicable showed similarly consistent positive associations.

In expression-matched random 5−gene panel analyses, the true TAM5 score consistently outperformed all 1,000 random panels for correlation with xCell macrophage scores (empirical P< 0.001 in each cohort), and the median benchmark−score differences between TAM−rich and TAM−low tumors were likewise greater than those observed for any random panel. Phenotype-label permutation analyses also showed that the observed benchmark-score differences between TAM-rich and TAM-low tumors were unlikely to arise from randomly assigned group labels. These findings reduce the possibility that the benchmark associations were merely a consequence of arbitrary five-gene scoring or random binary classification, although they remain transcriptomic benchmark analyses rather than functional validation.

### 3.6 Tissue-level validation in the local cohort supported a macrophage-enriched, hypoxia-linked niche

**Figure 5:**
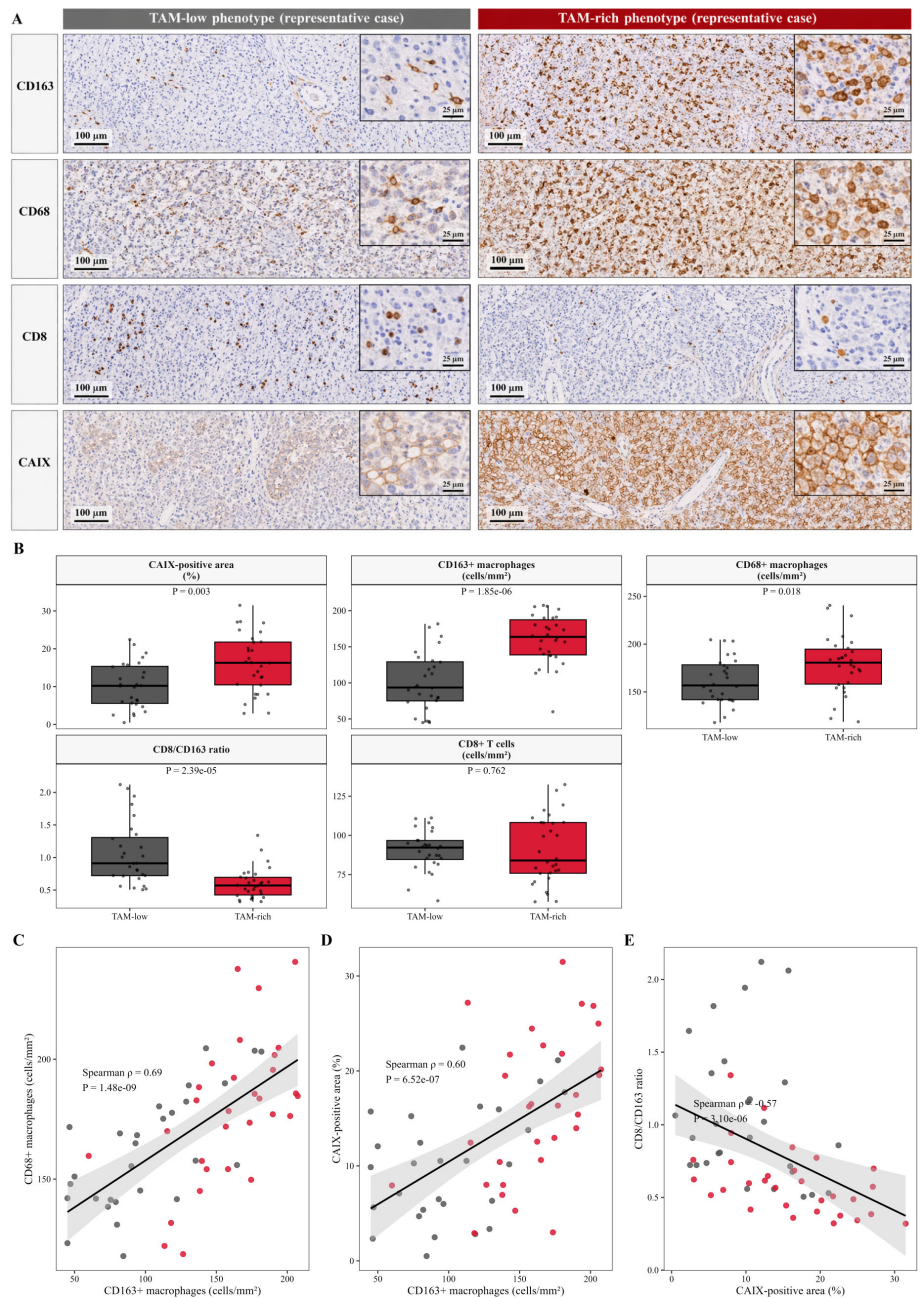
Local histopathological correlation of a macrophage-enriched, hypoxia-linked tissue contexture. **(A)** Representative immunohistochemical staining of CD163, CD68, CD8, and CAIX in serial sections from a macrophage-low representative case and a CD163-defined macrophage-enriched representative case. The macrophage-enriched case showed increased CD163-positive and CD68-positive macrophage infiltration together with stronger CAIX-related hypoxia signal. CD8-positive T cells were not uniformly absent, but appeared relatively restricted compared with the prominent macrophage-rich background. Main images are shown at intermediate magnification, with higher-magnification insets highlighting representative cellular staining patterns. Scale bars: 100 microm in the main panels and 25 microm in the insets. **(B)** Quantitative digital pathology analysis comparing CAIX-positive area, CD163-positive macrophage density, CD68-positive macrophage density, CD8/CD163 ratio, and CD8-positive T-cell density between macrophage-low and CD163-defined macrophage-enriched local tumors. Macrophage-associated and hypoxia-related tissue features were increased in macrophage-enriched tumors, whereas the CD8/CD163 ratio was reduced. CD8-positive T-cell density did not differ significantly at the global case level. **(C)** Correlation between CD163-positive macrophage density and CD68-positive macrophage density. **(D)** Correlation between CD163-positive macrophage density and CAIX-positive area. **(E)** Correlation between CAIX-positive area and the CD8/CD163 ratio.

Local histopathological analysis provided a parallel tissue-level correlate of the transcriptomic phenotype but did not directly validate transcriptome-defined patient assignment because matched RNA-seq data were not available in the FFPE cohort (**Figure 5**; **Supplementary Table 3**). Representative immunohistochemical staining showed that the CD163-defined macrophage-enriched representative case had more prominent CD163-positive and CD68-positive macrophage infiltration, together with stronger CAIX staining, than the macrophage-low representative case (**Figure 5A**). CD8-positive T cells remained detectable in both cases, but appeared relatively less prominent in the macrophage-enriched background.

Case-level quantitative digital pathology was concordant with the representative images (**Figure 5B**). Compared with macrophage-low tumors, the CD163-defined macrophage-enriched tissue group showed higher CAIX-positive area (P = 0.003), higher CD163-positive macrophage density (P = 1.85 × 10^-6), and higher CD68-positive macrophage density (P = 0.018). By contrast, CD8-positive T-cell density did not differ significantly at the global case level (P = 0.762). However, the CD8/CD163 ratio was significantly lower in macrophage-enriched tumors (P = 2.39 × 10^-5), indicating a relative shift toward macrophage predominance rather than absolute loss of CD8-positive cells. Because this ratio is mathematically driven by CD163-positive cell density, it is interpreted as a supportive indicator of immune balance rather than an independent tissue feature.

Correlation analyses further supported the internal coherence of the tissue-level findings. CD163-positive macrophage density correlated positively with CD68-positive macrophage density (Spearman rho = 0.69, P = 1.48 × 10^-9; **Figure 5C**) and with CAIX-positive area (Spearman rho = 0.60, P = 6.52 × 10^-7; **Figure 5D**). In contrast, CAIX-positive area correlated inversely with the CD8/CD163 ratio (Spearman rho = -0.57, P = 3.10 × 10^-6; **Figure 5E**). Together, these findings support a histologically recognizable macrophage-enriched, hypoxia-linked tissue analogue of the transcriptome-defined TAM-rich phenotype.

### 3.7 Sensitivity analyses supported robustness of the phenotype definition

Additional sensitivity analyses were performed to assess whether the phenotype definition was disproportionately driven by any single component gene, with special attention to SPP1 because it is not macrophage-exclusive and may also be expressed in malignant or hypoxia-associated tumor regions (**Supplementary Figure 4**; **Supplementary Table 5**). In leave-one-gene-out analyses, agreement with the original binary phenotype assignment remained high across cohorts, ranging from 90.4% to 98.4%. The corresponding continuous recalculated scores also remained highly correlated with the original 5-gene score (Spearman rho = 0.964-0.998), including the 4-gene panel excluding SPP1.

Continuous-score Cox models preserved the overall adverse direction of association across cohorts, but effect sizes were modest and not all reformulations reached nominal significance in every dataset. The 4-gene panel excluding SPP1 attenuated some associations without reversing the overall biological pattern, indicating that the phenotype was not driven by a single marker and was not solely dependent on median dichotomization.

## 4 Discussion

In this multi-cohort study, we characterized a compact transcriptome-defined TAM-rich phenotype in glioblastoma using a predefined 5-gene macrophage-oriented panel. Several findings were consistent across the discovery and validation transcriptomic datasets and were paralleled by local tissue observations. First, the phenotype was biologically coherent and reproducible across TCGA, CGGA_693, and CGGA_325, with stable enrichment of macrophage-associated genes, including CD163, MSR1, MRC1, CSF1R, SPP1, VSIG4, MARCO, and TREM1. Second, the phenotype was accompanied by a hypoxia-related component, reflected by increased CA9 expression at the transcriptomic level and stronger CAIX staining at the protein/tissue level. Third, supportive immune infiltration analyses, external macrophage benchmark analyses, and local histopathology did not support a purely immune-cold interpretation. Instead, they supported a macrophage-dominant immune contexture in which T-cell-related signals remained detectable but were relatively less prominent than the myeloid component. Finally, the phenotype showed only modest and cohort-dependent survival association, suggesting that its main value lies in biological stratification rather than in serving as a stand-alone prognostic classifier.

These results fit well with the current view that macrophages and microglia are not a minor accessory compartment in glioblastoma, but a dominant and plastic component of the tumor microenvironment. Reviews and translational studies have consistently shown that glioblastoma-associated macrophages/microglia can support tumor growth, invasion, immune suppression, vascular remodeling, and treatment resistance (7, 9, 10). At the same time, single-cell and spatial studies have made it increasingly clear that the myeloid compartment in glioblastoma is heterogeneous in ontogeny, localization, and activation state, and that simple M1/M2 binning is biologically insufficient (13, 15, 42). In that context, our decision to define a compact macrophage-oriented phenotype rather than to claim a canonical “M2 program” appears justified. The phenotype captured a stable myeloid-biased transcriptomic gradient, but one that should be interpreted as an immunoregulatory TAM-rich state rather than as a direct surrogate for a single macrophage subtype.

A notable strength of the present analysis is that the phenotype was anchored in markers with clear biological relevance in glioma-associated macrophages. CD163 has repeatedly been associated with higher-grade glioma, IDH-wildtype biology, and unfavorable immune contexture (30). CSF1R is one of the best-established macrophage-regulatory pathways in glioblastoma, and preclinical work showed that CSF1R inhibition could alter macrophage polarization states and restrain glioma progression (31). SPP1 is particularly important because it has emerged from both bulk and single-cell analyses as a marker of a suppressive, functionally active macrophage state rather than merely a marker of macrophage abundance (12, 32). Likewise, MARCO, VSIG4, and TREM1 are consistent with a macrophage program linked to scavenger function, immune regulation, and inflammatory remodeling rather than to simple cell counting alone (15, 33). This likely explains why the phenotype behaved as a biologically stable tissue-program signature rather than as a narrow histiocytic burden index.

The repeated association with CA9/CAIX is also biologically plausible and, in our view, central to the interpretation of this phenotype. Hypoxia is a defining ecological pressure in glioblastoma and has broad effects on invasion, metabolism, angiogenesis, stem-like behavior, and treatment resistance (18, 19, 22). Importantly, hypoxia also shapes immune composition and can favor suppressive myeloid programs. Prior experimental work showed that hypoxia can intensify macrophage–glioma interactions and enhance invasion-related signaling (20). More recent transcriptomic and single-cell frameworks have linked hypoxic regions and mesenchymal-like tumor states to macrophage-rich microenvironments (24, 25). Our data do not prove a causal hypoxia-to-TAM pathway, but they do support a consistent co-association between macrophage enrichment and hypoxia-related tissue context across public and local datasets. For that reason, we think the most accurate reading is not “macrophages alone,” but a macrophage-enriched, hypoxia-linked immune niche.

An equally important finding is what the phenotype did not represent. Although macrophage-associated markers were strongly enriched, T-cell-related signals were not uniformly suppressed across cohorts, and local histopathology showed that CD8-positive cells were still detectable in CD163-defined macrophage-enriched tumors. What changed more consistently was the balance between CD8 and macrophage compartments, reflected locally by the reduced CD8/CD163 ratio. This distinction matters. In glioblastoma, ineffective antitumor immunity is often driven less by absolute absence of T cells than by spatial exclusion, functional exhaustion, and suppressive myeloid control (26, 27). Our orthogonal infiltration analyses therefore support a more nuanced model in which TAM-rich tumors are not simply devoid of lymphocytes, but instead show a myeloid-dominant immune contexture likely to be unfavorable for effective T-cell function. This interpretation is more consistent with current glioblastoma immunobiology than a binary immune-hot/immune-cold framework.

The additional benchmark and negative-control analyses also help address the concern that macrophage enrichment might be a circular consequence of defining the phenotype with macrophage-associated genes. xCell macrophage-related scores and published no-TAM5 macrophage/TAM signatures were concordant with the TAM5 score across all three transcriptomic cohorts. More importantly, expression-matched random 5-gene panel analyses and phenotype-label permutation analyses showed that the observed benchmark associations were stronger than expected under random-panel or random-label null settings. These analyses do not constitute functional validation, but they reduce the likelihood that the observed immune-context signal is merely an artefact of arbitrary five-gene scoring or random dichotomization.

The prognostic results require cautious interpretation. Using the recalculated five-gene score–derived binary phenotype and the same adjusted Cox modeling strategy across cohorts, the original five-gene phenotype was not statistically significant in TCGA, CGGA_693, or CGGA_325. Continuous-score models showed only modest associations, with nominal significance limited to the CGGA cohorts. These findings argue against presenting the TAM-rich phenotype as an independent prognostic factor. Instead, the phenotype is best viewed as a microenvironmental stratifier that may complement, rather than replace, established clinical and molecular classifiers. This interpretation is also biologically plausible because glioblastoma outcome is determined by multiple interacting variables, including age, performance status, extent of resection, MGMT methylation, treatment intensity, tumor cell-intrinsic programs, and the immune microenvironment (2, 3).

We also consider the local histopathological cohort to be an important translational correlate of this work, while recognizing its logical limits. Because matched transcriptomic data were unavailable, the local FFPE samples do not form a closed validation loop for the transcriptome-defined phenotype. Instead, they show that a CD163-defined macrophage-enriched tissue state is accompanied by higher CD68-positive macrophage density, stronger CAIX signal, and a lower CD8/CD163 ratio, while global CD8 density itself is not significantly different. This tissue-level pattern parallels the transcriptomic and supportive infiltration results and makes the phenotype easier to interpret biologically: it is not just a computational label, but a histologically recognizable microenvironmental state.

Another point in favor of the phenotype is its internal robustness, although robustness should not be confused with external clinical portability. Compact gene-panel phenotypes can justifiably be criticized if they depend excessively on one dominant component or if their effect appears only after arbitrary dichotomization. Our sensitivity analyses argue against both concerns. Leave-one-gene-out reformulations preserved high agreement with the original phenotype assignment, and the recalculated continuous scores remained highly correlated with the original 5-gene score. Excluding SPP1 attenuated but did not reverse the overall pattern, which is informative rather than problematic. It suggests that SPP1 contributes meaningful biological information, as expected from prior work, but does not single-handedly drive the phenotype (32). Likewise, within-cohort z-score standardized continuous-score models support the use of the score as a quantitative immune-state measure and reduce dependence on raw platform-specific expression scales.

Several limitations should be acknowledged. First, the transcriptomic analyses were based on bulk tumor data and therefore cannot fully separate malignant-cell programs from microenvironmental composition. Single-cell studies have shown that glioblastoma myeloid states vary by ontogeny and region, and that macrophage-rich regions can couple to tumor cell state transitions (13, 25). Our study cannot resolve those cellular interactions directly. Second, the supportive immune infiltration, xCell, and published-signature benchmark analyses remain transcriptomic analyses and cannot fully substitute for single-cell, spatial, or functional macrophage validation. Third, the external datasets differed in platform and sample composition, which may partly explain the weaker or stronger survival signals across cohorts. Finally, the local FFPE cohort provided histopathological correlation rather than mechanistic proof and did not include matched RNA-seq data. These questions are better addressed in future spatial and functional studies.

Even with these limitations, the study has several practical implications. Methodologically, it shows that a short, biologically curated macrophage-oriented panel can recover a reproducible and interpretable phenotype across independent glioblastoma cohorts. Biologically, it supports a model in which macrophage enrichment, hypoxia, and relative T-cell disadvantage are linked but not reducible to one another. Translationally, it suggests that patients with a TAM-rich phenotype may be relevant candidates for strategies aimed at myeloid remodeling, hypoxia-targeted intervention, or rational combinations designed to reduce myeloid suppression and improve T-cell efficacy (9, 31, 34). Whether this phenotype can predict therapeutic response remains unknown, but our results provide a coherent basis for testing that question prospectively.

In summary, we identified a compact transcriptome-defined TAM-rich phenotype in glioblastoma that was reproducible at the transcriptomic level across TCGA and two independent CGGA cohorts and paralleled by macrophage-enriched, hypoxia-linked histopathological features in a local FFPE cohort. The phenotype was not simply equivalent to a purely immune-cold state, and its prognostic contribution was modest rather than dominant. Continuous-score, threshold-portability, external benchmark, and negative-control analyses support its interpretation as a biologically grounded framework for stratifying macrophage-dominant immune contextures in glioblastoma.

## Data Availability

The raw data supporting the conclusions of this article will be made available by the authors, without undue reservation.
