# Supplementary material for: A transcriptome-defined TAM-rich phenotype identifies a macrophage-enriched, hypoxia-linked immune contexture in glioblastoma: multi-cohort transcriptomic validation and local histopathological correlation
Source: Front Immunol. 2026 Jun 17;17:1871268. doi: 10.3389/fimmu.2026.1871268 (PMC13318709; doi:10.3389/fimmu.2026.1871268)
Supplement: Supplementary file 1 [file DataSheet1.docx]

**Supplementary Materials**


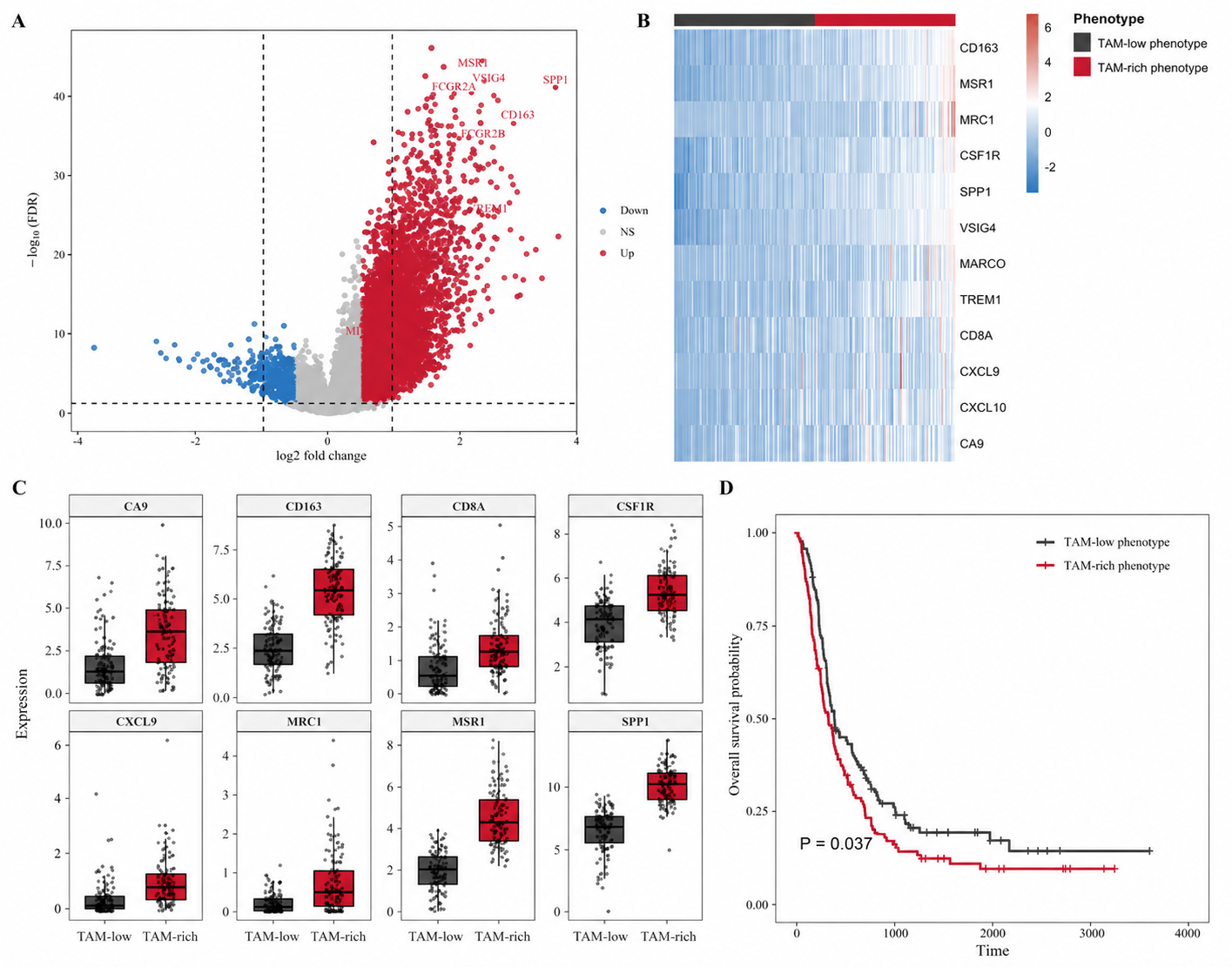


**Supplementary Figure S1. Detailed external validation of the TAM-rich phenotype in CGGA_693.**

(A) Volcano plot of differentially expressed genes between tumors with the TAM-rich phenotype and those with the TAM-low phenotype.

(B) Heatmap of representative macrophage-, T-cell-, and hypoxia-related markers.

(C) Boxplots comparing representative marker expression between groups.

(D) Kaplan-Meier analysis of overall survival according to phenotypic status. These analyses demonstrate that the core biological characteristics of the phenotype identified in TCGA were reproducible in CGGA_693; survival associations are exploratory and should not be interpreted as evidence that the phenotype is an independent prognostic factor.


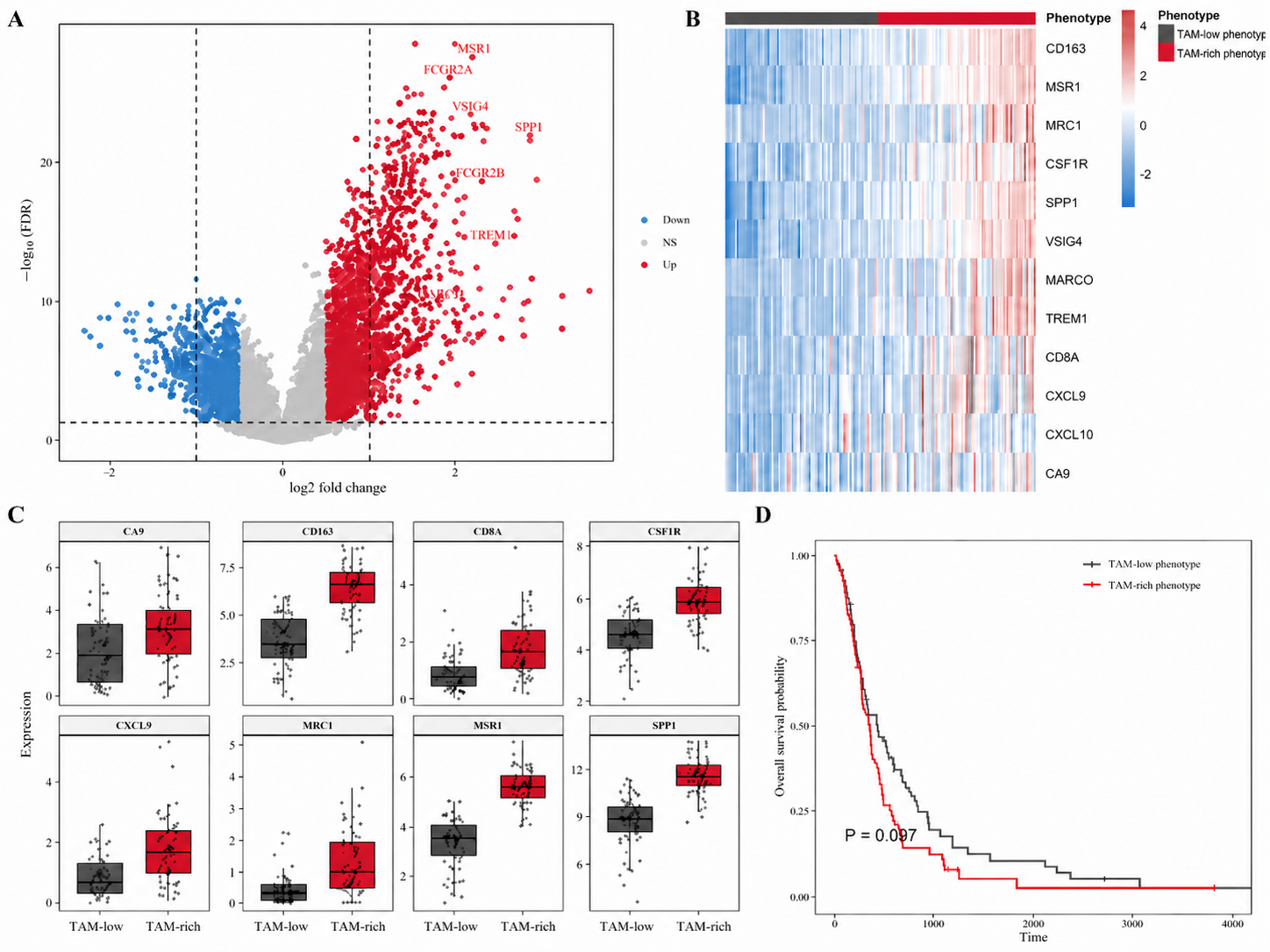


**Supplementary Figure S2. Detailed external validation of the TAM-rich phenotype in CGGA_325.**

(A) Volcano plot of differentially expressed genes between tumors with the TAM-rich phenotype and those with the TAM-low phenotype.

(B) Heatmap of representative macrophage-, T-cell-, and hypoxia-related markers.

(C) Boxplots comparing representative marker expression between groups.

(D) Kaplan-Meier analysis of overall survival according to phenotypic status. Although the survival association was weaker and the adjusted binary Cox model was not statistically significant, the direction of marker expression changes remained consistent, supporting biological rather than prognostic robustness across independent datasets.


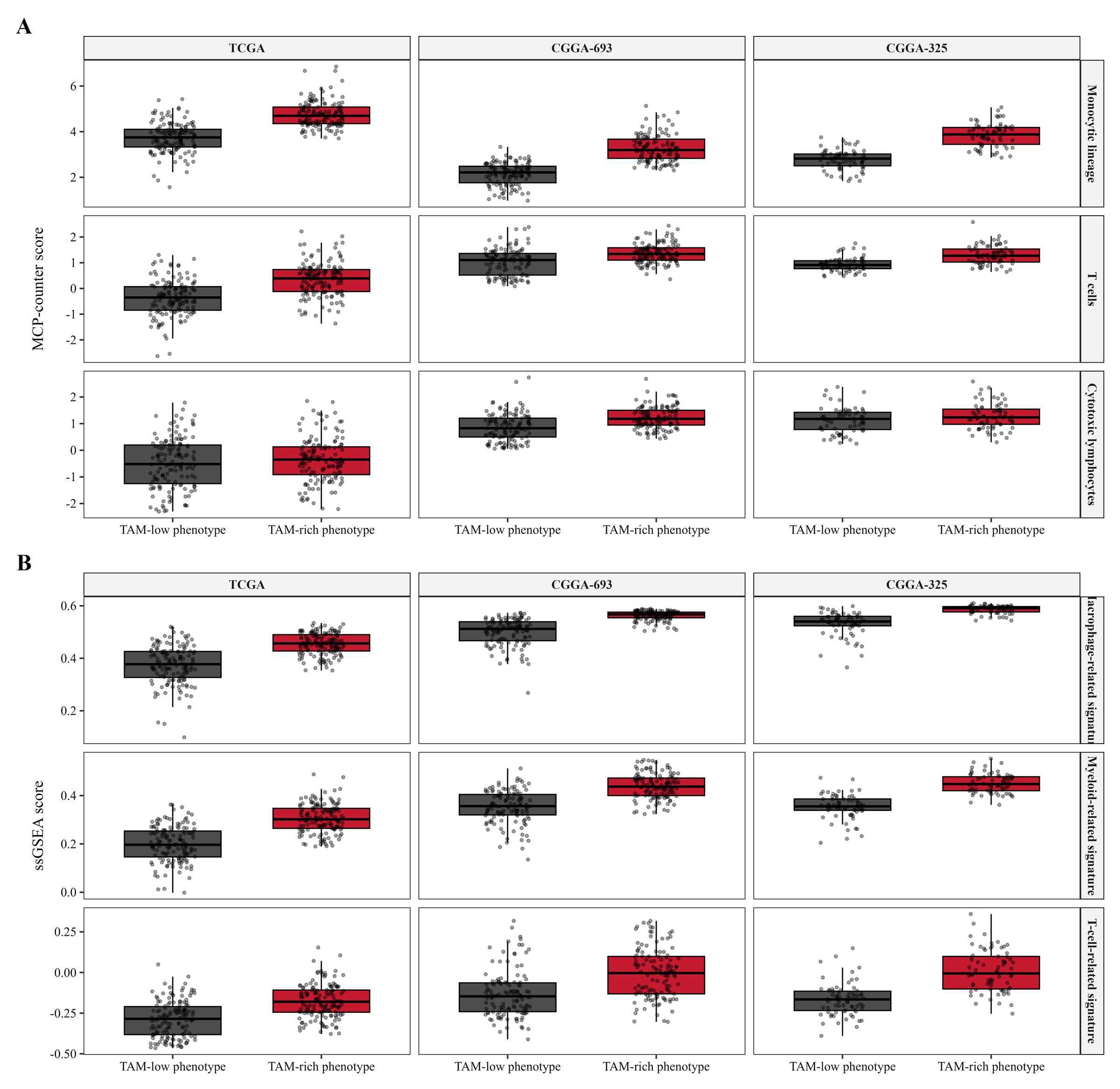


**Supplementary Figure S3. Supportive immune infiltration analyses across cohorts.**

(A) MCP-counter scores for monocytic lineage, T cells, and cytotoxic lymphocytes stratified by phenotypic status in TCGA, CGGA_693, and CGGA_325.

(B) Supportive ssGSEA/GSVA scores for macrophage-, myeloid-, and T-cell-related signatures across the same cohorts. These gene sets were intentionally defined without the core phenotype-defining macrophage genes (CD163, MSR1, MRC1, CSF1R, and SPP1) and without CD8A/CXCL9/CXCL10 to reduce direct circularity. Because the remaining genes remain lineage-related and co-regulated, these analyses are interpreted as supportive immune-context analyses rather than fully independent validation.


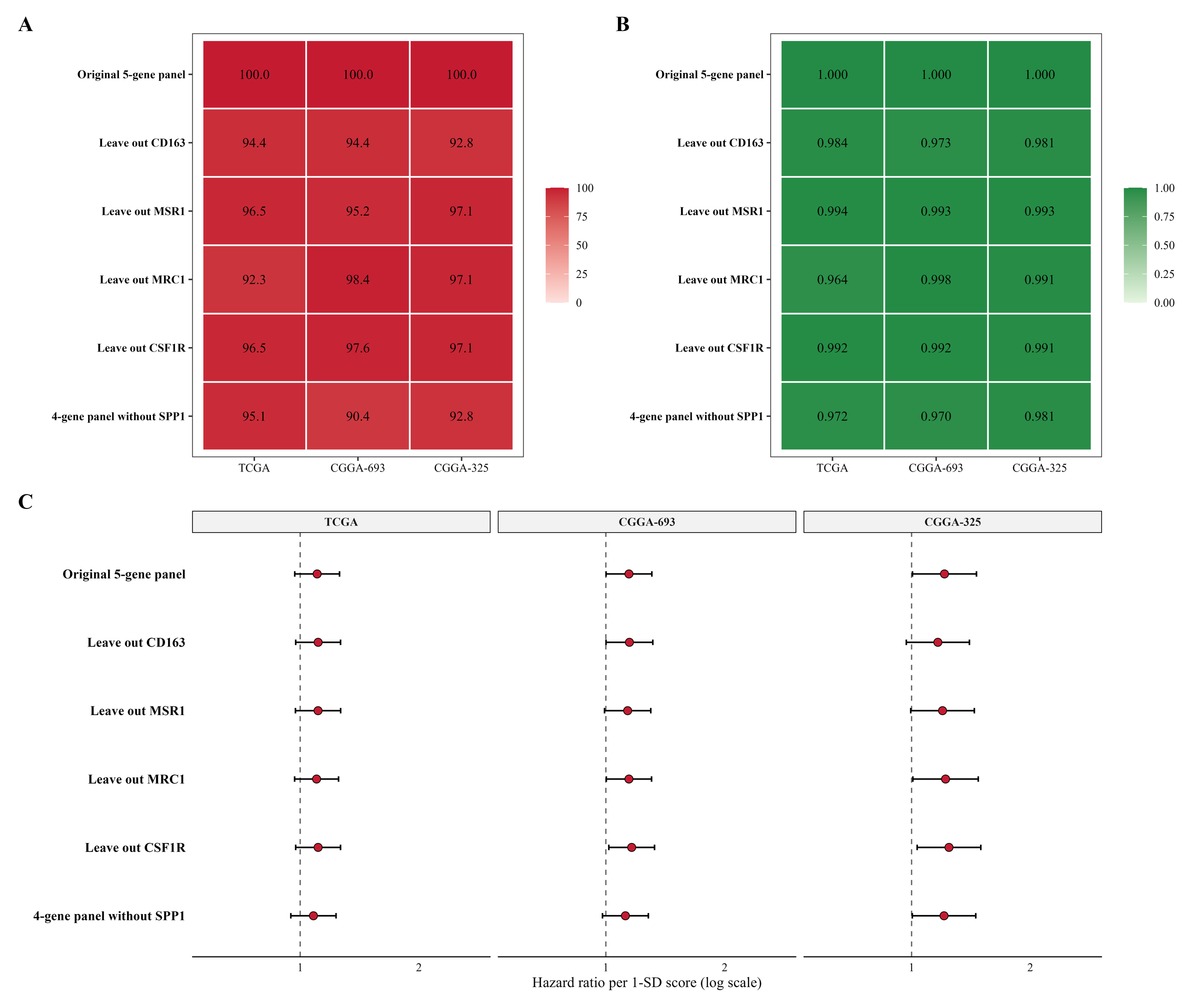


**Supplementary Figure S4. Sensitivity analyses of the transcriptome-defined TAM-rich phenotype.**

(A) Heatmap showing agreement in binary phenotype assignment between the original 5-gene panel and alternative phenotype formulations across TCGA, CGGA_693, and CGGA_325. Leave-one-gene-out recalculations and the 4-gene panel excluding SPP1 all showed high concordance with the original phenotype definition.

(B) Heatmap showing Spearman correlations between the original continuous 5-gene phenotype score and recalculated continuous scores from the alternative formulations across the three cohorts. Score-level correlations remained uniformly high, indicating strong structural stability of the phenotype definition.

(C) Forest plots showing hazard ratios and 95% confidence intervals from Cox proportional hazards models using the recalculated continuous phenotype score per 1-standard-deviation increase in TCGA, CGGA_693, and CGGA_325. Across cohorts, the overall direction of association remained stable despite modest variation in effect size and statistical significance between individual reformulations; these analyses are exploratory.


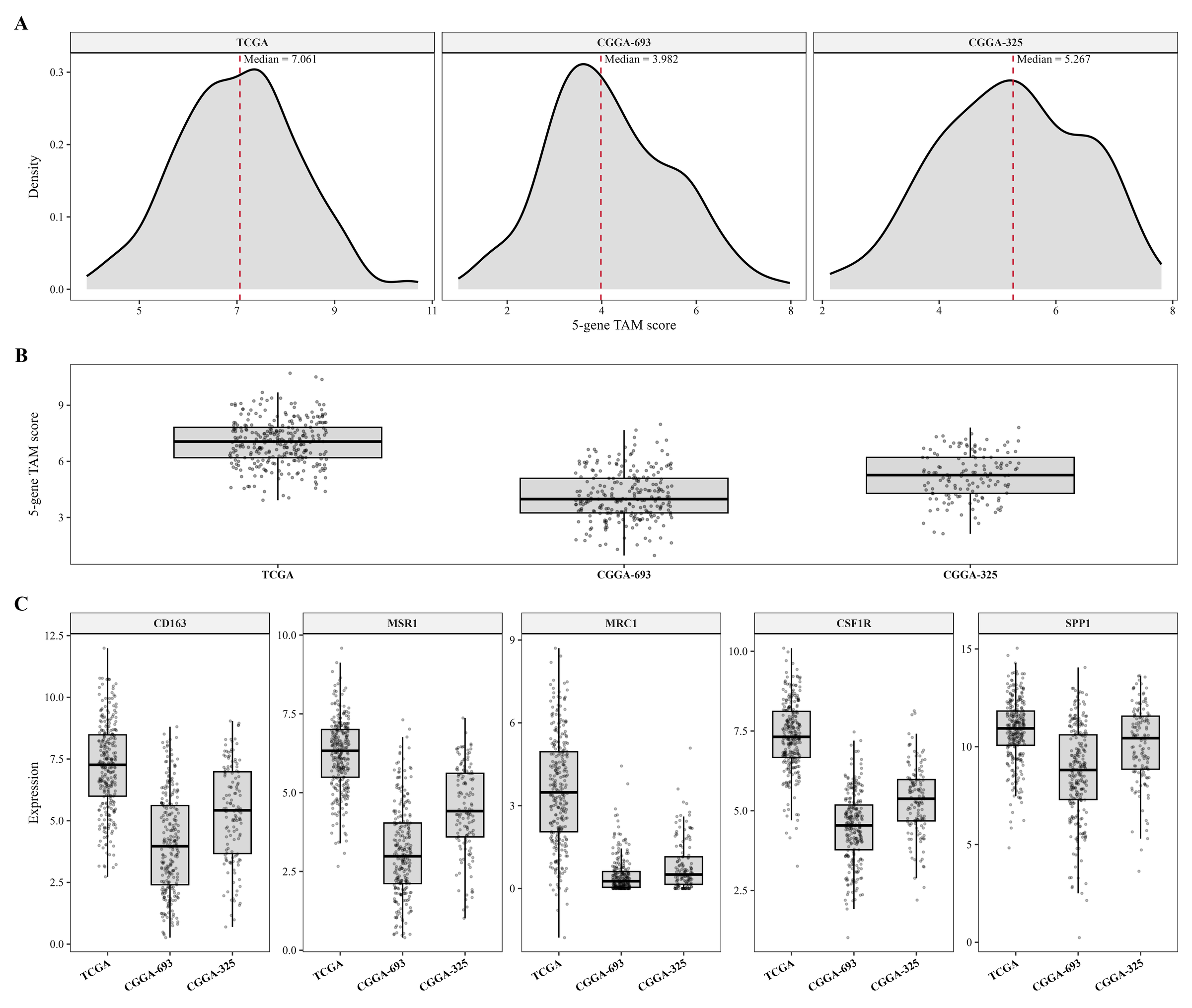


**Supplementary Figure S5. Distribution of the continuous 5-gene TAM score and its component genes across TCGA and CGGA cohorts.**

The continuous 5-gene TAM score was calculated for each sample as the mean expression of CD163, MSR1, MRC1, CSF1R, and SPP1. (A) Density plots showing the distribution of the continuous 5-gene TAM score in TCGA, CGGA-693, and CGGA-325. The red dashed vertical line indicates the cohort-specific median cutoff used for secondary median-based classification; median values are rounded in the figure, and exact cutoffs are reported in Supplementary Table S6. (B) Boxplots with overlaid individual data points showing the distribution of the continuous 5-gene TAM score across the three cohorts. (C) Boxplots with overlaid individual data points showing the expression distribution of each score-defining gene across cohorts. In boxplots, the center line indicates the median, boxes indicate the interquartile range, whiskers extend to 1.5 times the interquartile range, and points represent individual samples. Because expression values were derived from different transcriptomic platforms, raw expression scales and median cutoffs should be interpreted within each cohort rather than directly transferred across cohorts.


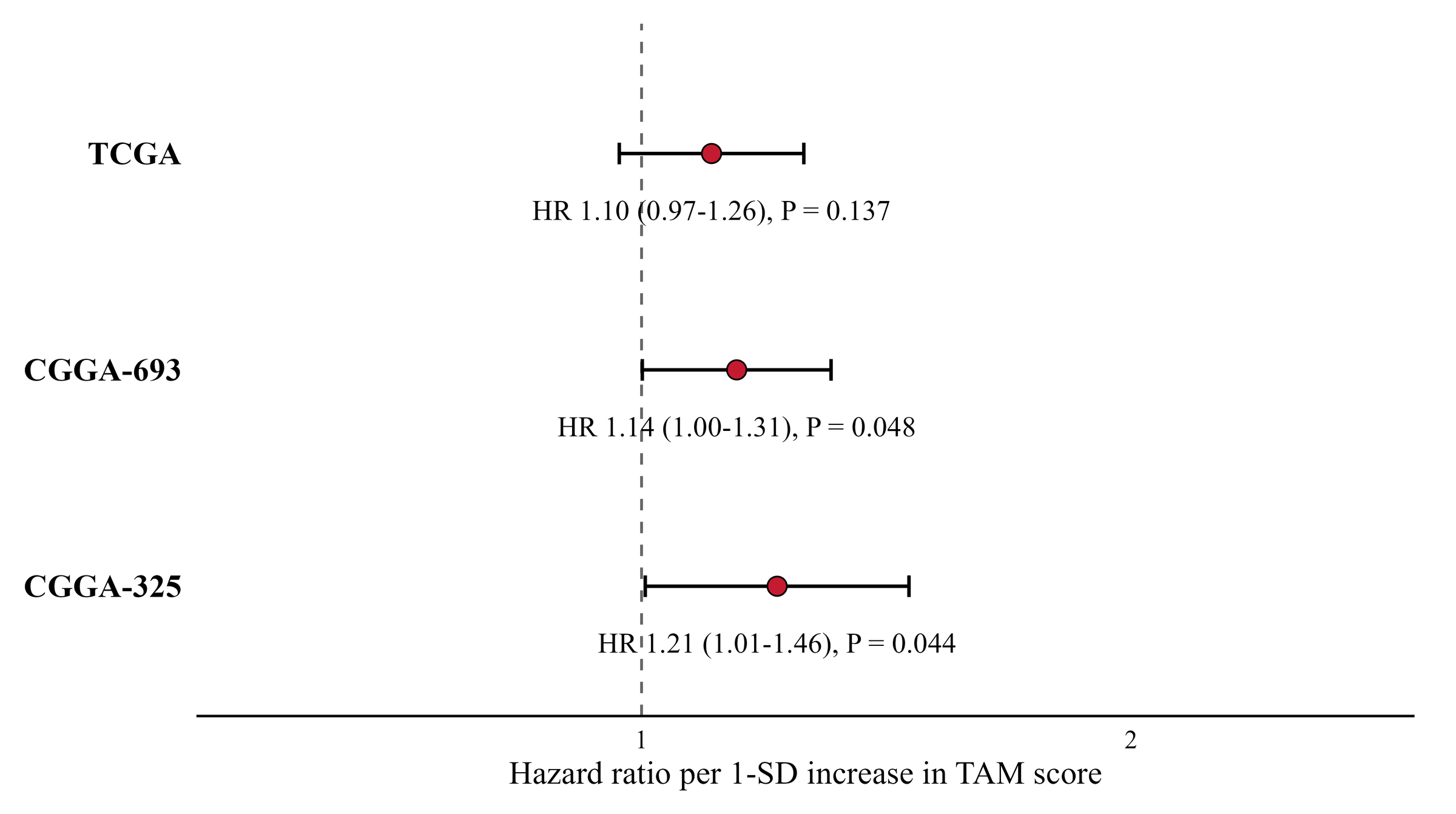


**Supplementary Figure S6. Survival association of the within-cohort z-score standardized continuous 5-gene TAM score.**

Forest plot showing Cox proportional hazards models for overall survival using the continuous 5-gene TAM score after within-cohort z-score standardization in TCGA, CGGA-693, and CGGA-325. The hazard ratio represents the relative change in overall survival risk per 1-SD increase in the 5-gene TAM score. Models were adjusted for available age and sex variables within each cohort. Points indicate hazard ratios, horizontal bars indicate 95% confidence intervals, and the vertical dashed line represents a hazard ratio of 1. The standardized continuous-score models showed modest positive associations with overall survival risk in CGGA-693 and CGGA-325, whereas the association did not reach statistical significance in TCGA. These analyses were performed to evaluate the continuous score while reducing dependence on raw platform-specific expression scales.


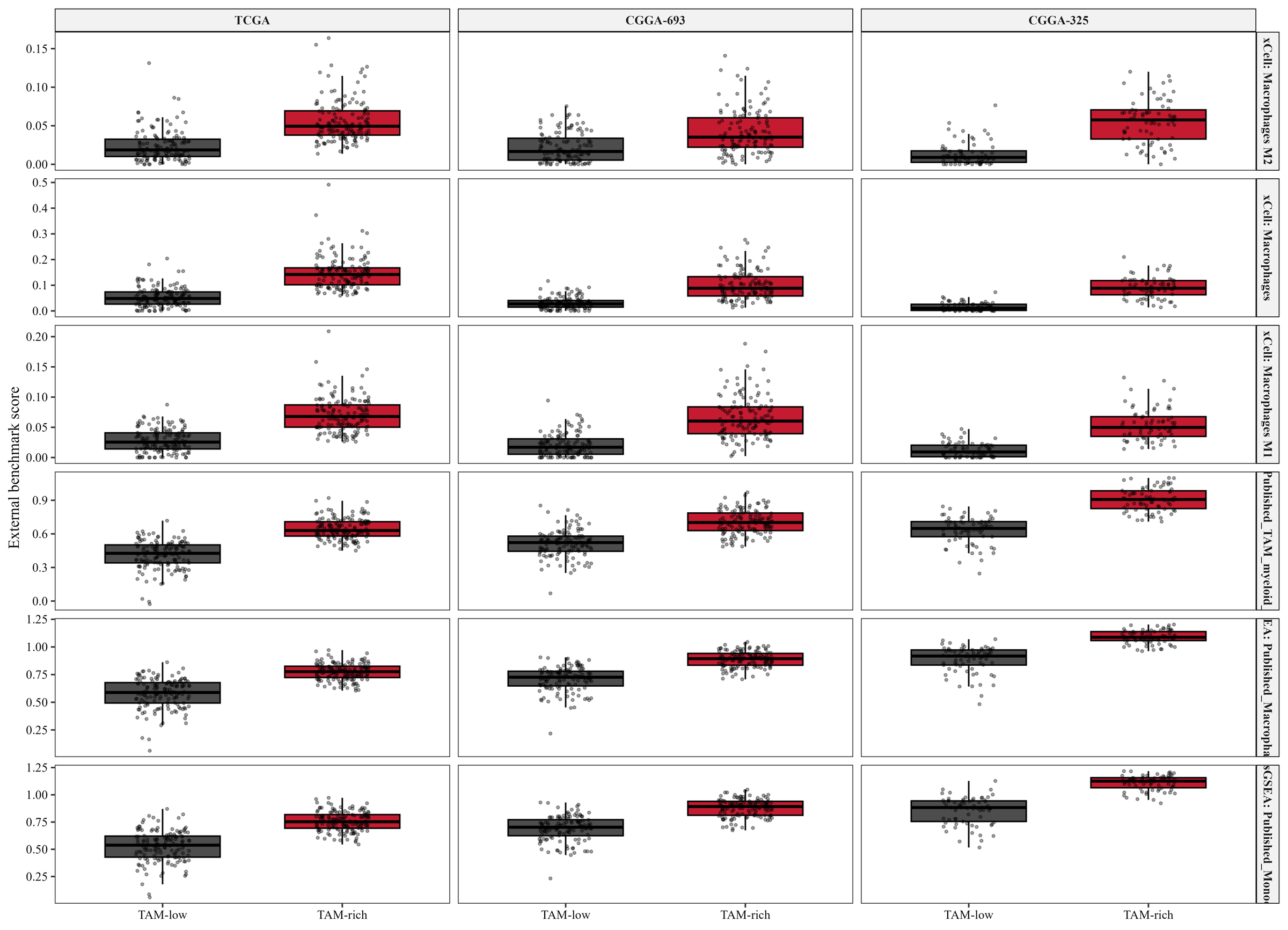


**Supplementary Figure S7. External macrophage-related benchmark scores according to the five-gene TAM phenotype across cohorts.**

Boxplots show external immune benchmark scores in TAM-low and TAM-rich tumors across TCGA, CGGA-693, and CGGA-325. The TAM phenotype was defined using the cohort-specific median of the continuous five-gene TAM score, calculated as the mean expression of CD163, MSR1, MRC1, CSF1R, and SPP1. External benchmark scores included xCell-derived macrophage-related scores (Macrophages, Macrophages M1, and Macrophages M2) and published macrophage/TAM-related ssGSEA signatures, with the five TAM-score-defining genes excluded from published benchmark signatures where applicable. Boxes indicate the interquartile range, center lines indicate medians, whiskers extend to 1.5 times the interquartile range, and points represent individual samples. These analyses were performed to assess whether the TAM5-defined phenotype was concordant with external macrophage-related immune benchmarks while reducing direct circularity.


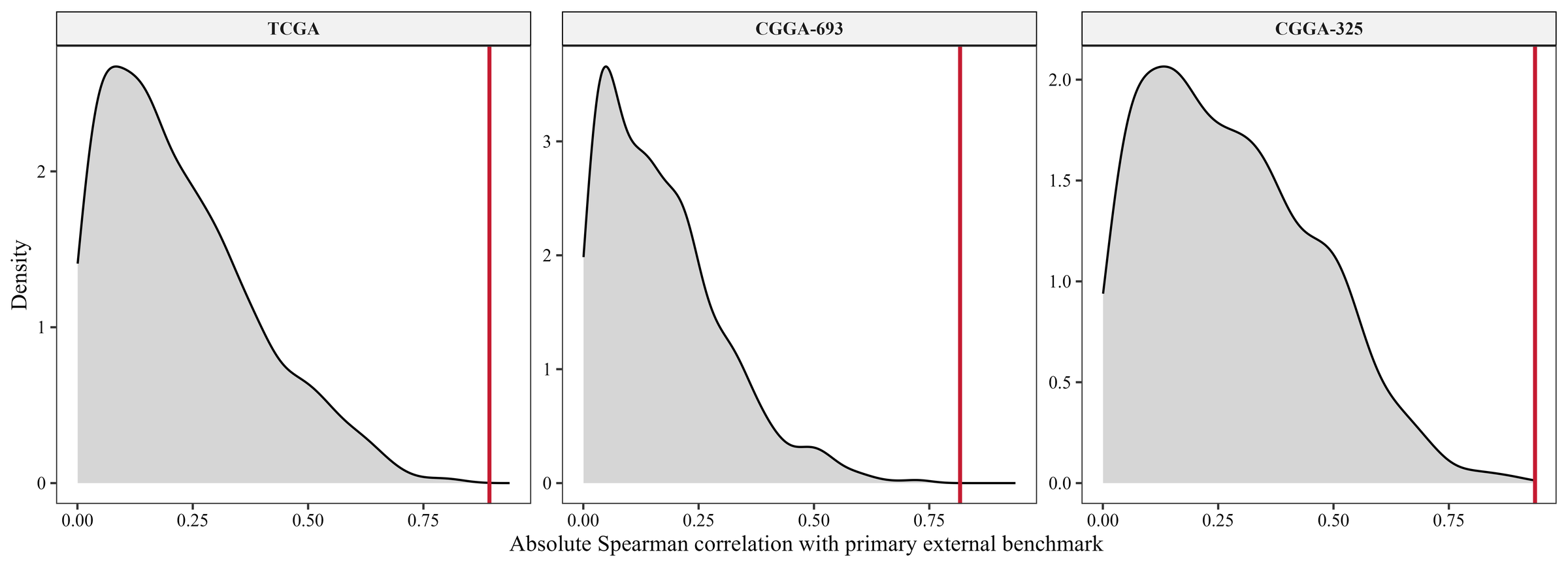


**Supplementary Figure S8. Negative-control analysis using expression-matched random five-gene panels.**

Density plots show the null distribution of absolute Spearman correlations between expression-matched random five-gene scores and the primary external macrophage/TAM benchmark in TCGA, CGGA-693, and CGGA-325. For each cohort, 1,000 random five-gene panels were sampled from expression-matched bins after excluding the five TAM-score-defining genes (CD163, MSR1, MRC1, CSF1R, and SPP1). The red vertical line indicates the observed absolute Spearman correlation between the true TAM5 score and the primary external benchmark. The observed TAM5 benchmark association was located at the extreme right tail of the null distribution in all three cohorts, indicating that the association was stronger than expected for random expression-matched five-gene panels. The corresponding empirical P values are reported in Supplementary Table S7.


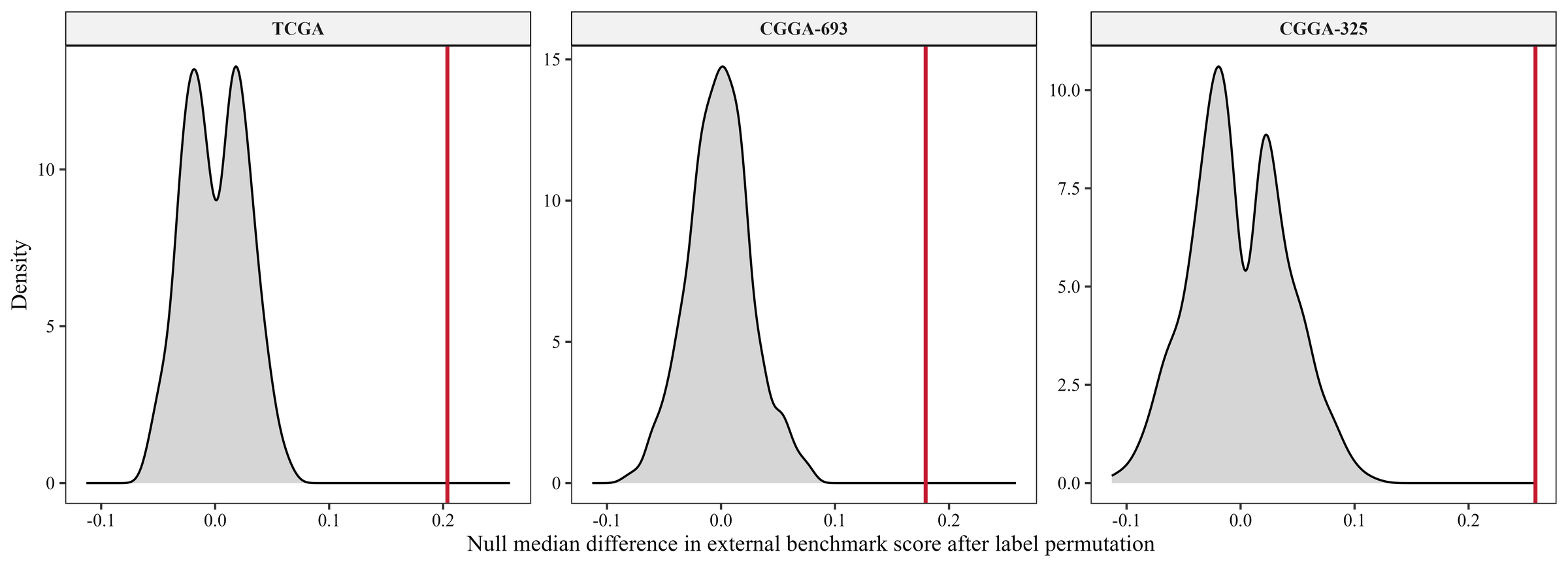


**Supplementary Figure S9. Negative-control analysis using phenotype-label permutation.**

Density plots show the null distribution of median differences in the primary external macrophage/TAM benchmark score after random permutation of TAM-rich and TAM-low labels within each cohort. Group sizes were preserved during permutation. The red vertical line indicates the observed median difference in benchmark score between the true TAM-rich and TAM-low groups. In TCGA, CGGA-693, and CGGA-325, the observed benchmark-score difference was located far outside the permutation-derived null distribution, suggesting that the observed enrichment was unlikely to result from random binary labeling. The corresponding empirical P values are reported in Supplementary Table S7.

**Supplementary Tables**

**Supplementary Table S1. Baseline characteristics of CGGA_693.**

| **Variable** | **Level** | **Overall** | **TAM-low** | **TAM-rich** | **P value** |
| --- | --- | --- | --- | --- | --- |
| No. of cases |  | 249 | 124 | 125 |  |
| Age, years |  | 50.0 (41.0-60.0) | 48.5 (34.8-58.2) | 50.0 (42.0-61.0) | 0.077 |
| Overall survival time, days |  | 378.0 (221.0-768.0) | 402.0 (250.0-834.0) | 344.5 (174.0-699.5) | 0.029 |
| Sex |  |  |  |  | 0.898 |
|  | Female | 102 (41.0) | 50 (40.3) | 52 (41.6) |  |
|  | Male | 147 (59.0) | 74 (59.7) | 73 (58.4) |  |
| Vital status/event |  |  |  |  | 0.084 |
|  | Alive/censored | 40 (16.1) | 25 (20.2) | 15 (12.0) |  |
|  | Dead | 198 (79.5) | 93 (75.0) | 105 (84.0) |  |
|  | Missing/unknown | 11 (4.4%) | 6 (4.8%) | 5 (4.0%) |  |

Continuous variables are presented as median (IQR), and categorical variables are presented as n (%). P values were calculated using the Wilcoxon rank-sum test for continuous variables and Fisher’s exact test for categorical variables, as appropriate.

**Supplementary Table S2. Baseline characteristics of CGGA_325.**

| **Variable** | **Level** | **Overall** | **TAM-low** | **TAM-rich** | **P value** |
| --- | --- | --- | --- | --- | --- |
| No. of cases |  | 139 | 69 | 70 |  |
| Age, years |  | 48.0 (39.5-56.0) | 43.0 (36.0-54.0) | 51.0 (42.0-59.0) | 0.003 |
| Overall survival time, days |  | 348.0 (209.0-614.0) | 422.0 (213.5-787.5) | 346.5 (195.8-542.0) | 0.187 |
| Sex |  |  |  |  | 0.600 |
|  | Female | 51 (36.7) | 27 (39.1) | 24 (34.3) |  |
|  | Male | 88 (63.3) | 42 (60.9) | 46 (65.7) |  |
| Vital status/event |  |  |  |  | 0.776 |
|  | Alive/censored | 13 (9.4) | 7 (10.1) | 6 (8.6) |  |
|  | Dead | 124 (89.2) | 60 (87.0) | 64 (91.4) |  |
|  | Missing/unknown | 2 (1.4%) | 2 (2.9%) | 0 (0.0%) |  |

Continuous variables are presented as median (IQR), and categorical variables are presented as n (%). P values were calculated using the Wilcoxon rank-sum test for continuous variables and Fisher’s exact test for categorical variables, as appropriate.

**Supplementary Table S3. Baseline and digital pathology characteristics of the local histopathological cohort.**

| **Variable** | **Level** | **Overall** | **TAM-low** | **TAM-rich** | **P value** |
| --- | --- | --- | --- | --- | --- |
| No. of cases |  | 58 | 28 | 30 |  |
| Age, years |  | 61.0 (53.0–68.0) | 60.5 (52.0–67.0) | 62.0 (54.0–69.0) | 0.438 |
| CD163+ macrophages, cells/mm2 |  | 136.9 (93.3-171.8) | 93.5 (75.0-129.2) | 163.7 (138.7-187.4) | < 0.001 |
| CD68+ macrophages, cells/mm2 |  | 171.8 (148.4-187.7) | 156.8 (141.8-178.3) | 180.6 (158.1-194.7) | 0.018 |
| CD8+ T cells, cells/mm2 |  | 89.7 (78.2-104.4) | 92.2 (84.6-96.8) | 84.0 (75.9-108.2) | 0.762 |
| CAIX-positive area, % |  | 12.5 (7.0-18.6) | 10.2 (5.6-15.4) | 16.3 (10.5-21.8) | 0.003 |
| CD8/CD163 ratio |  | 0.7 (0.5-1.0) | 0.9 (0.7-1.3) | 0.6 (0.4-0.7) | < 0.001 |

Continuous variables are presented as median (interquartile range), and categorical variables are presented as n (%). The local cohort included 58 cases from the Sichuan Cancer Hospital. CD163, CD68, and CD8 were quantified as positive cells/mm²; CAIX was quantified as positive area percentage. The CD8/CD163 ratio was calculated from case-level cell densities.

**Supplementary Table S4. Orthogonal immune signature gene sets used for GSVA/ssGSEA analyses.**

| **Signature** | **Genes included** | **Genes intentionally excluded** | **Purpose** |
| --- | --- | --- | --- |
| Macrophage orthogonal | AIF1, APOE, C1QA, C1QB, C1QC, FCER1G, TYROBP, LAPTM5, LST1, CTSB | CD163, MSR1, MRC1, CSF1R, SPP1 | Orthogonal macrophage-related enrichment |
| Myeloid orthogonal | TYROBP, FCER1G, AIF1, LST1, SAT1, CTSB, LAPTM5, FCN1, HK3, CFD | CD163, MSR1, MRC1, CSF1R, SPP1 | Orthogonal broader myeloid-related enrichment |
| T-cell orthogonal | CD2, CD3D, CD3E, TRBC1, TRBC2, LTB, IL7R, TRAT1, CCL5, NKG7 | CD8A, CXCL9, CXCL10 | Orthogonal T-cell-related enrichment |

Note: The macrophage and myeloid signatures intentionally excluded the five phenotype-defining genes CD163, MSR1, MRC1, CSF1R, and SPP1. The T-cell-related signature intentionally excluded CD8A, CXCL9, and CXCL10 to reduce circularity in the orthogonal validation.

**Supplementary Table S5. Sensitivity analyses of the transcriptome-defined TAM-rich phenotype.**

| **Cohort** | **Variant** | **Genes included** | **No. of genes** | **Score rho vs original** | **Phenotype agreement (%)** | **High-phenotype prevalence (%)** | **HR (binary)** | **P (binary)** | **HR per 1-SD score** | **P (continuous)** |
| --- | --- | --- | --- | --- | --- | --- | --- | --- | --- | --- |
| TCGA | Original 5-gene panel | CD163, MSR1, MRC1, CSF1R, SPP1 | 5 | 1.000 | 100.0 | 50.2 | 1.06 (0.81-1.37) | 0.683 | 1.10 (0.97-1.26) | 0.137 |
| TCGA | Leave out CD163 | MSR1, MRC1, CSF1R, SPP1 | 4 | 0.984 | 94.4 | 50.2 | 1.14 (0.87-1.48) | 0.342 | 1.11 (0.97-1.27) | 0.116 |
| TCGA | Leave out MSR1 | CD163, MRC1, CSF1R, SPP1 | 4 | 0.994 | 96.5 | 50.2 | 1.04 (0.80-1.35) | 0.750 | 1.11 (0.97-1.27) | 0.121 |
| TCGA | Leave out MRC1 | CD163, MSR1, CSF1R, SPP1 | 4 | 0.964 | 92.3 | 50.2 | 1.11 (0.85-1.43) | 0.448 | 1.10 (0.97-1.25) | 0.143 |
| TCGA | Leave out CSF1R | CD163, MSR1, MRC1, SPP1 | 4 | 0.992 | 96.5 | 50.2 | 1.06 (0.82-1.38) | 0.654 | 1.11 (0.97-1.27) | 0.117 |
| TCGA | 4-gene panel without SPP1 | CD163, MSR1, MRC1, CSF1R | 4 | 0.972 | 95.1 | 50.2 | 1.01 (0.78-1.31) | 0.917 | 1.08 (0.95-1.23) | 0.250 |
| CGGA-693 | Original 5-gene panel | CD163, MSR1, MRC1, CSF1R, SPP1 | 5 | 1.000 | 100.0 | 50.2 | 1.32 (1.00-1.75) | 0.054 | 1.14 (1.00-1.31) | 0.048 |
| CGGA-693 | Leave out CD163 | MSR1, MRC1, CSF1R, SPP1 | 4 | 0.973 | 94.4 | 50.2 | 1.28 (0.96-1.70) | 0.091 | 1.15 (1.00-1.32) | 0.049 |
| CGGA-693 | Leave out MSR1 | CD163, MRC1, CSF1R, SPP1 | 4 | 0.993 | 95.2 | 50.2 | 1.28 (0.96-1.71) | 0.086 | 1.14 (0.99-1.30) | 0.065 |
| CGGA-693 | Leave out MRC1 | CD163, MSR1, CSF1R, SPP1 | 4 | 0.998 | 98.4 | 50.2 | 1.30 (0.98-1.73) | 0.066 | 1.14 (1.00-1.31) | 0.046 |
| CGGA-693 | Leave out CSF1R | CD163, MSR1, MRC1, SPP1 | 4 | 0.992 | 97.6 | 50.2 | 1.31 (0.99-1.74) | 0.059 | 1.16 (1.02-1.33) | 0.027 |
| CGGA-693 | 4-gene panel without SPP1 | CD163, MSR1, MRC1, CSF1R | 4 | 0.970 | 90.4 | 50.2 | 1.23 (0.92-1.64) | 0.156 | 1.12 (0.98-1.28) | 0.095 |
| CGGA-325 | Original 5-gene panel | CD163, MSR1, MRC1, CSF1R, SPP1 | 5 | 1.000 | 100.0 | 50.4 | 1.33 (0.93-1.91) | 0.122 | 1.21 (1.01-1.46) | 0.044 |
| CGGA-325 | Leave out CD163 | MSR1, MRC1, CSF1R, SPP1 | 4 | 0.981 | 92.8 | 50.4 | 1.33 (0.92-1.90) | 0.127 | 1.17 (0.97-1.40) | 0.103 |
| CGGA-325 | Leave out MSR1 | CD163, MRC1, CSF1R, SPP1 | 4 | 0.993 | 97.1 | 50.4 | 1.39 (0.97-1.99) | 0.076 | 1.20 (1.00-1.44) | 0.056 |
| CGGA-325 | Leave out MRC1 | CD163, MSR1, CSF1R, SPP1 | 4 | 0.991 | 97.1 | 50.4 | 1.30 (0.90-1.86) | 0.158 | 1.22 (1.01-1.48) | 0.042 |
| CGGA-325 | Leave out CSF1R | CD163, MSR1, MRC1, SPP1 | 4 | 0.991 | 97.1 | 50.4 | 1.38 (0.96-1.99) | 0.082 | 1.24 (1.03-1.50) | 0.021 |
| CGGA-325 | 4-gene panel without SPP1 | CD163, MSR1, MRC1, CSF1R | 4 | 0.981 | 92.8 | 50.4 | 1.36 (0.95-1.96) | 0.095 | 1.21 (1.00-1.45) | 0.045 |

Note: The original phenotype was defined using the mean log-scale expression of CD163, MSR1, MRC1, CSF1R, and SPP1. Within each cohort, the binary TAM-rich phenotype was defined using the cohort-specific median of the corresponding score. Leave-one-gene-out variants were recalculated by excluding one gene at a time. The 4-gene panel without SPP1 is shown explicitly because of the biological relevance of SPP1 to macrophage functional state. Continuous analyses were modeled per 1-SD increase in the corresponding score. Cox models were adjusted for available age and sex variables within each cohort.

**Supplementary Table S6A. Distribution of the continuous 5-gene TAM score and exact cohort-specific median cutoffs.**

| **Cohort** | **No. with score** | **Mean** | **SD** | **Median cutoff** | **Q1** | **Q3** | **IQR** | **Range** | **TAM-low, n** | **TAM-rich, n** |
| --- | --- | --- | --- | --- | --- | --- | --- | --- | --- | --- |
| TCGA | 285 | 7.022 | 1.226 | 7.060516 | 6.193 | 7.817 | 1.624 | 3.922 to 10.712 | 142 | 143 |
| CGGA-693 | 249 | 4.170 | 1.315 | 3.982328 | 3.243 | 5.095 | 1.852 | 0.966 to 7.979 | 124 | 125 |
| CGGA-325 | 139 | 5.221 | 1.231 | 5.267081 | 4.287 | 6.213 | 1.926 | 2.130 to 7.808 | 69 | 70 |

Note: The 5-gene TAM score was calculated as the mean expression of CD163, MSR1, MRC1, CSF1R, and SPP1. Median cutoffs are reported with six decimal places to allow exact reproducibility of the median-based binary classification. Because expression units differ across platforms, the cutoffs are cohort-specific and should not be directly transferred across cohorts.

**Supplementary Table S6B. Distribution of the component genes used to derive the 5-gene TAM score.**

| **Cohort** | **Gene** | **No. available** | **Mean** | **SD** | **Median** | **Q1** | **Q3** | **IQR** | **Range** |
| --- | --- | --- | --- | --- | --- | --- | --- | --- | --- |
| TCGA | CD163 | 285 | 7.182 | 1.784 | 7.264 | 5.995 | 8.480 | 2.485 | 2.729 to 11.991 |
| TCGA | MSR1 | 285 | 6.225 | 1.120 | 6.327 | 5.489 | 7.004 | 1.515 | 2.661 to 9.583 |
| TCGA | MRC1 | 285 | 3.542 | 1.985 | 3.481 | 2.053 | 4.952 | 2.899 | -1.781 to 8.705 |
| TCGA | CSF1R | 285 | 7.341 | 1.077 | 7.316 | 6.672 | 8.115 | 1.443 | 3.261 to 10.097 |
| TCGA | SPP1 | 285 | 10.823 | 1.629 | 10.939 | 10.075 | 11.826 | 1.751 | 4.830 to 15.039 |
| CGGA-693 | CD163 | 249 | 4.073 | 2.009 | 3.969 | 2.406 | 5.615 | 3.209 | 0.263 to 8.808 |
| CGGA-693 | MSR1 | 249 | 3.122 | 1.450 | 2.984 | 2.114 | 4.037 | 1.923 | 0.401 to 7.310 |
| CGGA-693 | MRC1 | 249 | 0.483 | 0.651 | 0.263 | 0.043 | 0.614 | 0.571 | 0.000 to 4.436 |
| CGGA-693 | CSF1R | 249 | 4.489 | 1.106 | 4.542 | 3.777 | 5.180 | 1.403 | 1.021 to 7.471 |
| CGGA-693 | SPP1 | 249 | 8.681 | 2.390 | 8.812 | 7.303 | 10.608 | 3.305 | 0.239 to 14.053 |
| CGGA-325 | CD163 | 139 | 5.325 | 1.974 | 5.423 | 3.671 | 6.983 | 3.313 | 0.696 to 9.041 |
| CGGA-325 | MSR1 | 139 | 4.480 | 1.385 | 4.415 | 3.596 | 5.615 | 2.019 | 1.014 to 7.369 |
| CGGA-325 | MRC1 | 139 | 0.830 | 0.911 | 0.506 | 0.151 | 1.147 | 0.997 | 0.000 to 5.085 |
| CGGA-325 | CSF1R | 139 | 5.330 | 1.080 | 5.376 | 4.681 | 5.977 | 1.296 | 2.198 to 8.134 |
| CGGA-325 | SPP1 | 139 | 10.142 | 1.960 | 10.439 | 8.850 | 11.564 | 2.714 | 3.631 to 13.627 |

Note: Expression distributions are shown for each score-defining gene within each cohort. These summaries are intended to make the contribution and scale of each component gene transparent.

**Supplementary Table S6C. Cox models using the continuous 5-gene TAM score.**

| **Cohort** | **Model** | **N** | **Events** | **HR (95% CI)** | **P** | **Covariates** |
| --- | --- | --- | --- | --- | --- | --- |
| TCGA | Raw continuous score | 285 | 232 | 1.08 (0.97-1.21) | 0.137 | Age and sex |
| CGGA-693 | Raw continuous score | 237 | 197 | 1.11 (1.00-1.23) | 0.048 | Age and sex |
| CGGA-325 | Raw continuous score | 137 | 124 | 1.17 (1.00-1.36) | 0.044 | Age and sex |
| TCGA | Within-cohort z-score | 285 | 232 | 1.10 (0.97-1.26) | 0.137 | Age and sex |
| CGGA-693 | Within-cohort z-score | 237 | 197 | 1.14 (1.00-1.31) | 0.048 | Age and sex |
| CGGA-325 | Within-cohort z-score | 137 | 124 | 1.21 (1.01-1.46) | 0.044 | Age and sex |

Note: Raw continuous-score models and within-cohort z-score standardized models are both shown. The z-score standardized model estimates the hazard ratio per 1-SD increase in the 5-gene TAM score and is more comparable across platforms. Models were adjusted for available age and sex variables within each cohort.

**Supplementary Table S7A. External immune benchmark scores according to the five-gene TAM-rich classification.**

| **Cohort** | **Benchmark** | **n low** | **n rich** | **Median low** | **Median rich** | **Delta median** | **Spearman rho with TAM5 score** | **P value** | **BH-FDR** |
| --- | --- | --- | --- | --- | --- | --- | --- | --- | --- |
| TCGA | xCell: Macrophages | 142 | 143 | 0.048 | 0.142 | 0.094 | 0.872 | 3.92e-36 | 3.82e-35 |
| TCGA | xCell: Macrophages M1 | 142 | 143 | 0.026 | 0.068 | 0.042 | 0.846 | 8.90e-32 | 3.86e-31 |
| TCGA | xCell: Macrophages M2 | 142 | 143 | 0.019 | 0.049 | 0.031 | 0.733 | 1.27e-26 | 2.91e-26 |
| TCGA | Monocyte/macrophage axis | 142 | 143 | 0.538 | 0.753 | 0.215 | 0.838 | 1.64e-34 | 1.01e-33 |
| TCGA | Macrophage Bindea-like (no TAM5) | 142 | 143 | 0.588 | 0.776 | 0.188 | 0.843 | 7.72e-34 | 3.76e-33 |
| TCGA | TAM myeloid immunosuppressive (no TAM5) | 142 | 143 | 0.426 | 0.630 | 0.204 | 0.893 | 2.82e-39 | 3.66e-38 |
| CGGA-693 | xCell: Macrophages | 124 | 125 | 0.027 | 0.088 | 0.061 | 0.853 | 1.94e-28 | 5.39e-28 |
| CGGA-693 | xCell: Macrophages M1 | 124 | 125 | 0.017 | 0.060 | 0.043 | 0.797 | 7.34e-27 | 1.79e-26 |
| CGGA-693 | xCell: Macrophages M2 | 124 | 125 | 0.017 | 0.035 | 0.019 | 0.535 | 1.95e-09 | 2.12e-09 |
| CGGA-693 | Monocyte/macrophage axis | 124 | 125 | 0.702 | 0.892 | 0.190 | 0.852 | 2.43e-29 | 7.29e-29 |
| CGGA-693 | Macrophage Bindea-like (no TAM5) | 124 | 125 | 0.727 | 0.894 | 0.167 | 0.878 | 2.43e-31 | 9.46e-31 |
| CGGA-693 | TAM myeloid immunosuppressive (no TAM5) | 124 | 125 | 0.522 | 0.702 | 0.179 | 0.817 | 1.57e-25 | 3.23e-25 |
| CGGA-325 | xCell: Macrophages | 69 | 70 | 0.011 | 0.088 | 0.078 | 0.921 | 2.40e-21 | 3.34e-21 |
| CGGA-325 | xCell: Macrophages M1 | 69 | 70 | 0.009 | 0.050 | 0.041 | 0.899 | 3.93e-20 | 5.28e-20 |
| CGGA-325 | xCell: Macrophages M2 | 69 | 70 | 0.009 | 0.058 | 0.049 | 0.797 | 1.75e-16 | 2.14e-16 |
| CGGA-325 | Monocyte/macrophage axis | 69 | 70 | 0.883 | 1.125 | 0.242 | 0.938 | 4.57e-22 | 7.74e-22 |
| CGGA-325 | Macrophage Bindea-like (no TAM5) | 69 | 70 | 0.918 | 1.088 | 0.170 | 0.933 | 1.08e-21 | 1.62e-21 |
| CGGA-325 | TAM myeloid immunosuppressive (no TAM5) | 69 | 70 | 0.649 | 0.907 | 0.259 | 0.937 | 4.76e-22 | 7.74e-22 |

Note: External benchmark scores included xCell macrophage-related scores and published macrophage/TAM-related ssGSEA signatures. For published benchmark signatures, the five TAM-score-defining genes (CD163, MSR1, MRC1, CSF1R, and SPP1) were excluded where applicable. P values were adjusted using the Benjamini-Hochberg method.

**Supplementary Table S7B. Expression-matched random 5-gene panel negative-control analysis.**

| **Cohort** | **Primary external benchmark** | **N** | **Observed \|rho\|** | **Empirical P for \|rho\|** | **Observed delta median** | **Empirical P for delta** | **No. random panels** |
| --- | --- | --- | --- | --- | --- | --- | --- |
| TCGA | TAM myeloid immunosuppressive (no TAM5) | 285 | 0.893 | 0.0010 | 0.204 | 0.0010 | 1,000 |
| CGGA-693 | TAM myeloid immunosuppressive (no TAM5) | 249 | 0.817 | 0.0010 | 0.179 | 0.0010 | 1,000 |
| CGGA-325 | TAM myeloid immunosuppressive (no TAM5) | 139 | 0.937 | 0.0010 | 0.259 | 0.002 | 1,000 |

Note: For each cohort, 1,000 random 5-gene panels were sampled from expression-matched bins after excluding CD163, MSR1, MRC1, CSF1R, and SPP1. The observed absolute Spearman correlation between the TAM5 score and the primary external benchmark was compared with the null distribution from random panels. Empirical P values were calculated as (1 + number of null statistics at least as extreme as observed)/(1 + number of valid random panels).

**Supplementary Table S7C. Phenotype-label permutation negative-control analysis.**

| **Cohort** | **Primary external benchmark** | **N** | **Observed delta median** | **Empirical P** | **No. permutations** |
| --- | --- | --- | --- | --- | --- |
| TCGA | TAM myeloid immunosuppressive (no TAM5) | 285 | 0.204 | 0.0010 | 1,000 |
| CGGA-693 | TAM myeloid immunosuppressive (no TAM5) | 249 | 0.179 | 0.0010 | 1,000 |
| CGGA-325 | TAM myeloid immunosuppressive (no TAM5) | 139 | 0.259 | 0.0010 | 1,000 |

Note: TAM-rich/TAM-low labels were randomly permuted within each cohort while preserving group sizes. The observed median difference in the primary external benchmark score was compared with the permutation-derived null distribution.
